# The Role of Estrogen Signaling Pathway and Targeted Therapy Exploration in Urological Tumors

**DOI:** 10.7150/ijbs.123812

**Published:** 2026-01-01

**Authors:** Cunzhen Ma, Lin Yang, Weijia Li, Wentai Shangguan, Wenxue Huang, Zhuohang Li, Boyuan Sun, Xunguo Yang, Haoxiang Xu, Zhibiao Li, Peidan Peng, Zongwei Wang, Peng Wu, Bisheng Cheng

**Affiliations:** 1 Department of Urology, Nanfang Hospital, Southern Medical University, Guangzhou, China.; 2 Department of Surgery, Division of Urology, Beth Israel Deaconess Medical Center, Harvard Medical School, Boston, MA, USA.; 3 Department of Urology, Second Affiliated Hospital of Naval Medical University, Shanghai, China.

**Keywords:** estrogen signaling, urological tumors, estrogen receptors, targeted therapy, ERα and ERβ, GPER

## Abstract

Estrogen signaling has emerged as a pivotal regulator in the development and progression of various cancers, including those of the urological system. While urological malignancies have traditionally been linked with androgen signaling, recent studies reveal a complex interplay where estrogen receptors—ERα, ERβ, and GPER—modulate critical cellular processes such as proliferation, apoptosis, and metastasis in these cancers. Here we show that estrogen receptors, through both genomic and non-genomic pathways, exert dual roles in either promoting or inhibiting tumor growth, making them both a challenge and a potential therapeutic target. These insights suggest that targeting estrogen receptor pathways could offer novel treatment strategies, especially for advanced or therapy-resistant urological tumors.Furthermore, understanding the molecular mechanisms through which estrogen receptors influence tumor progression could lead to the development of more specific and less toxic treatment options. The findings not only shift the paradigm of estrogen's role in cancer biology but also underscore the potential for personalized treatments, where estrogen receptor status could be used to tailor more effective and individualized therapeutic regimens. This review ushers in new possibilities for advancing urological oncology, where estrogen signaling may hold the key to overcoming current therapeutic challenges and improving clinical outcomes.

## 1. Introduction

The estrogen signaling pathway has long been recognized for its critical regulatory functions in reproductive physiology and hormone-dependent tumors such as breast cancer. Since the discovery of estrogen receptors (ERα and ERβ) as nuclear transcription factors in the mid-20th century, research has progressively unveiled their wide-ranging roles in cellular proliferation, differentiation, and survival. More recently, the identification of the membrane-associated G protein-coupled estrogen receptor (GPER) has expanded the landscape of estrogen biology, revealing rapid nongenomic effects that complement classical nuclear pathways. Historically, urological malignancies, including prostate, bladder, and renal cancers, were primarily associated with androgen signaling, and thus estrogen was not considered a central contributor to their pathogenesis. However, accumulating evidence over the past two decades has challenged this perspective, demonstrating that estrogen receptors are expressed in urological tissues and actively participate in tumor development and progression.

Emerging studies have highlighted the diverse and sometimes opposing roles of different estrogen receptor subtypes in urological cancers. For instance, ERβ often functions as a tumor suppressor by inhibiting proliferation and inducing apoptosis in prostate cancer cells, while ERα has been implicated in promoting tumor growth and invasion through activation of pathways such as PI3K/AKT and MAPK 1. Similarly, in bladder cancer, ERβ is highly expressed and is frequently associated with poor prognosis and disease progression, whereas ERα expression varies by tumor stage and may exhibit protective functions in certain contexts 2. In renal cell carcinoma, the role of estrogen signaling appears especially complex, with ERβ influencing cancer stemness, angiogenesis, and resistance to targeted therapies 3. These multifaceted effects underscore the necessity of understanding estrogen receptor biology in a nuanced, tumor-specific manner.

Given these insights, there is an urgent need to consolidate and critically appraise the expanding body of research on estrogen signaling in urological tumors. Despite significant advances, comprehensive reviews synthesizing molecular mechanisms, receptor subtype functions, and their translational relevance remain limited. This review seeks to fill this gap by systematically summarizing the current knowledge of estrogen-mediated pathways in prostate, bladder, and renal cancers. By integrating preclinical findings with clinical observations, we aim to elucidate how estrogen signaling contributes to tumor heterogeneity, therapeutic resistance, and disease progression. Moreover, we explore the emerging potential of estrogen receptor-targeted therapies as novel treatment strategies, particularly for advanced or refractory cases.

In the following sections, this review is organized to provide a coherent and logical exploration of the topic. First, we detail the molecular biology of estrogen receptors, including their genomic and nongenomic mechanisms of action. Next, we examine the specific roles of estrogen signaling in prostate cancer, bladder cancer, and renal cell carcinoma, highlighting both tumor-promoting and tumor-suppressive functions across receptor subtypes. We then discuss current and emerging therapeutic strategies targeting estrogen pathways, evaluating preclinical and clinical evidence supporting their application. Finally, we consider future perspectives and research directions, emphasizing the need for personalized approaches based on receptor expression profiles and tumor molecular characteristics. This structure ensures a consistent progression from fundamental mechanisms to translational implications, providing a comprehensive understanding of estrogen signaling in urological oncology.

## 2. Biological Effects of Estrogen on Urological Tumors

### 2.1 Overview of Estrogen Receptor Signaling Pathways

#### 2.1.1 Functions of ERα and ERβ and Their Expression in Urological Tumors

ERα and ERβ are widely distributed across multiple organs in the human body, including the breast, brain, cardiovascular system, urogenital tract, and skeletal system. By binding to estrogens such as E2, these receptors exert diverse physiological functions within these tissues. In preclinical models of various endocrine malignancies, such as breast, ovarian, and prostate cancers, the roles of ERα and ERβ differ significantly. In prostate tissues, ERβ is predominantly localized in epithelial cells, whereas ERα is primarily found in stromal cells 4. Furthermore, an increasing body of evidence indicates that estrogen-mediated ER signaling is closely associated with the initiation and progression of urothelial carcinomas, particularly in the luminal subtype of muscle-invasive bladder cancer, where ER activation plays a pivotal role.

**Figure 1:**
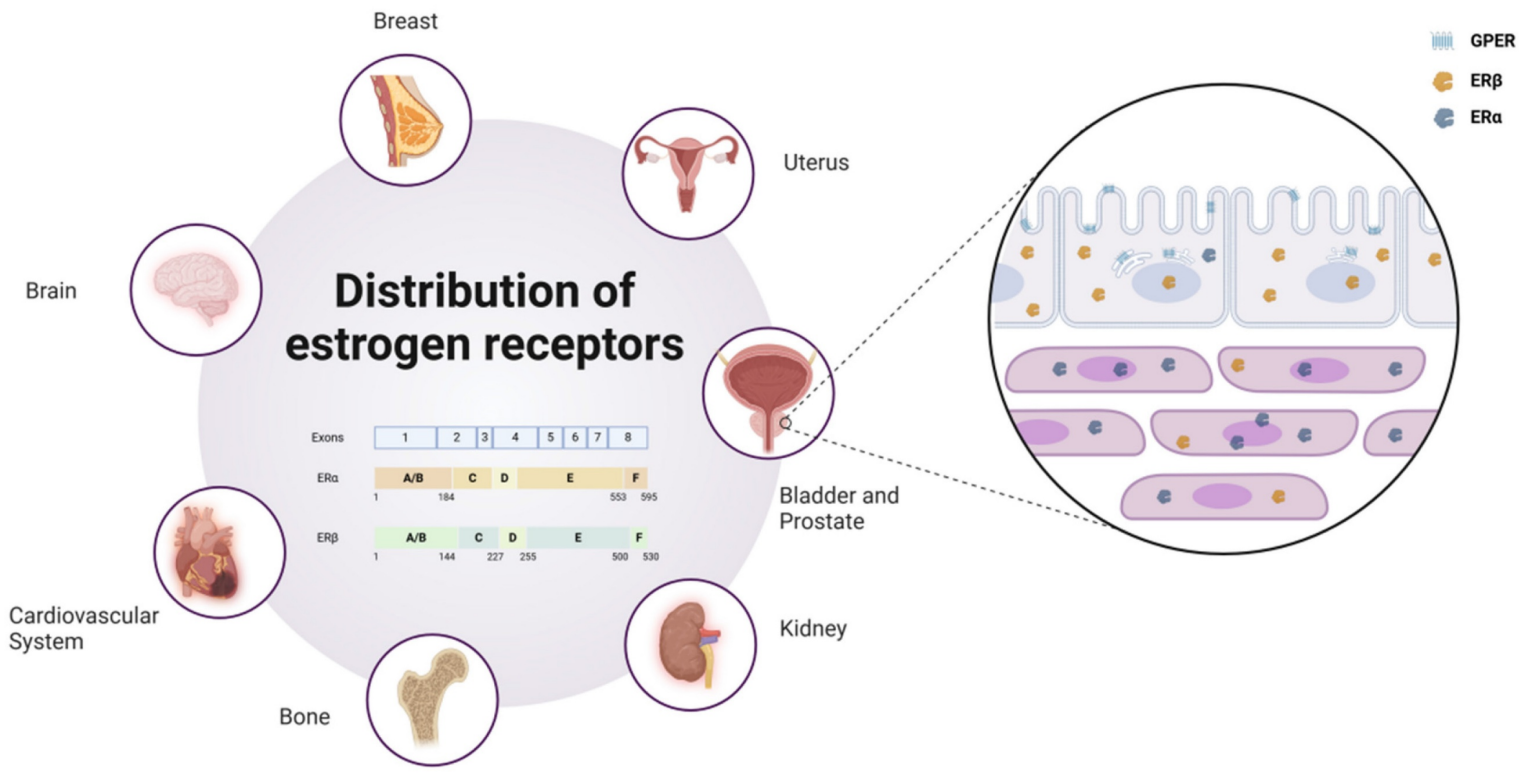
** Distribution and subtypes of estrogen receptors.** Estrogen receptors are widely distributed in various human organs, such as the Uterus, Breast, Brain, Cardiovascular System, Bone, Kidney, Bladder, and Prostate. In prostate tissue, ERβ is primarily localized in epithelial cells, while ERα is concentrated in stromal cells, and GPER is distributed across the cell membrane and endoplasmic reticulum. Schematic representation of estrogen receptor (ER) subtypes. ER is encoded by eight exons. The exons correspond to the structural domains in ER that are color-coded and labeled (A-F). Estrogen receptor alpha (ERα) consists of 595 amino acids, whereas estrogen receptor beta (ERβ) is composed of 530 amino acids.

Another key estrogen receptor, GPER, is primarily localized to the endoplasmic reticulum within cells 5. Its signaling relies on various heterotrimeric G proteins, such as Gαs, Gαi, Gβγ, and Gαq/11, which subsequently activate downstream cascades. These pathways include the generation of cAMP and the activation of signaling molecules such as EGFR, PKA, and CREB 6. Through these mechanisms, GPER plays a critical regulatory role in processes such as cell proliferation, differentiation, and survival. As depicted in Figure 1, estrogen receptors (ERα, ERβ, and GPER) show distinct expression patterns across tissues.

#### 2.1.2 Cellular Biological Effects of Estrogen Receptor Activation

ER regulate gene transcription by translocating to the nucleus and binding to specific response elements, resulting in changes in gene expression. This mechanism, known as the genomic or nuclear effect, typically exhibits a time delay, taking hours or even days to manifest. However, estrogens can also exert their biological activity through mechanisms that do not involve direct binding to DNA. This process, independent of gene transcription or protein synthesis, is referred to as the nongenomic or non-nuclear effect. Nongenomic effects are generally mediated by membrane-associated ER, which act much more rapidly, occurring within seconds to minutes. These responses often involve intracellular second messengers, such as calcium ion flux, cyclic adenosine monophosphate (cAMP) regulation, and signaling cascades involving MAPK and tyrosine kinases. Additionally, endoplasmic reticulum-associated ER can regulate gene expression via other mechanisms without directly interacting with DNA.

**Figure 2:**
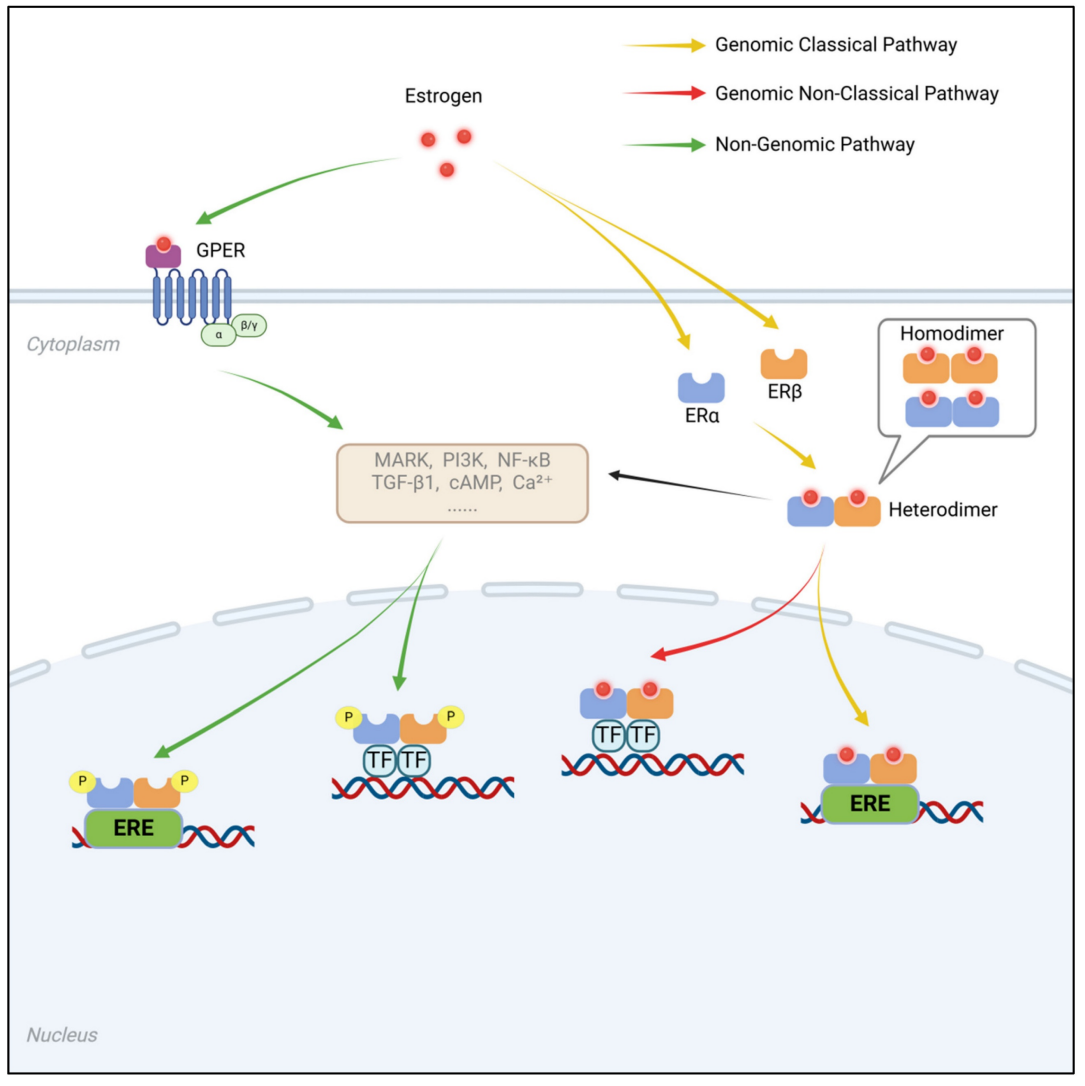
** Estrogen receptor-mediated genomic and non-genomic signaling pathways.** The mechanism by which estrogen regulates intracellular signal transduction and gene expression through different receptor pathways. It is broadly divided into three signaling pathways: the genomic classical pathway, the genomic non-classical pathway, and the non-genomic pathway. a. Genomic Classical Pathway (Yellow arrows): Estrogen binds to ERα or ERβ within the cell nucleus, forming a homodimer or heterodimer, which then binds to ERE (Estrogen Response Elements) on DNA, activating gene transcription. b. Genomic Non-Classical Pathway (Red arrows): After estrogen binds to ERα or ERβ, the receptors can interact with other transcription factors (TFs), influencing gene expression without direct dependence on ERE binding. c. Non-Genomic Pathway (Green arrows): Estrogen activates downstream signaling pathways such as MAPK, PI3K, NF-κB, TGF-β1, cAMP, and Ca²⁺ through GPER on the cell membrane, facilitating rapid cellular signaling. Additionally, ERα and ERβ are also involved in non-genomic signaling pathways, indirectly affecting gene expression through phosphorylation and protein interactions.

By binding to estrogens, ER play critical roles in the development, proliferation, migration, and survival of target cells. In females, both ERα and ERβ are essential for maintaining normal ovarian function. ERα, in particular, is crucial for the physiological functions of the uterus, breast development, and skeletal health. In males, although ER are not required for the normal development of reproductive organs, ERα is indispensable for male fertility. Furthermore, ERβ appears to exert protective effects against the progression of breast cancer, prostate cancer, and colorectal cancer, highlighting its potential value in cancer prevention. These findings demonstrate that both the genomic and nongenomic effects of ER have significant regulatory roles in a wide range of physiological and pathological processes. The genomic and non-genomic mechanisms of estrogen receptor signaling are summarized in Figure 2.

### 2.2 Estrogen and Prostate Cancer

#### 2.2.1 Expression and Regulatory Roles of ERα and ERβ in Prostate Cancer

Prostate cancer (PCa) is a hormone-dependent malignancy where estrogens play a critical role in initiation and progression. Estrogen receptors mediate estrogen signaling through ligand binding and receptor dimerization, with dimers binding to estrogen response elements (EREs) in target genes to regulate transcription. ERβ is highly expressed in prostate epithelial cells 7, while ERα is predominantly localized in stromal cells. The G protein-coupled estrogen receptor, located in the plasma membrane 8 and endoplasmic reticulum 5, contributes to tumorigenesis and metastasis by activating distinct signaling pathways.

##### 2.2.1.1 ERβ in Prostate Cancer

ERα generally promotes proliferation, inflammation, and migration, while ERβ has anti-proliferative, tumor-suppressive effects. Loss of ERβ is associated with progression to castration-resistant prostate cancer (CRPC) 9. ERβ expression is high in benign prostatic hyperplasia (BPH) and low Gleason score PCa, but decreases with higher Gleason scores, correlating with tumor progression 10. Interestingly, ERβ expression re-emerges in metastatic PCa, suggesting its potential as a therapeutic target 11.

ERβ suppresses PCa cell proliferation by regulating key signaling pathways. For example, in PC3 and 22RV1 cells, ERβ reduces oncogenic factors like Skp2 and c-Myc while upregulating cell cycle inhibitors like p21 and p27 12. ERβ also inhibits EGFR nuclear translocation, a process that enhances chemotherapy resistance, while promoting nuclear localization of PTEN, a tumor suppressor gene 13,14. During PCa progression, loss of ERβ is associated with nuclear EGFR accumulation, increased proliferative markers (e.g., PCNA), and loss of PTEN 14. Another mechanism of ERβ's anti-proliferative effects involve promoting HIF-1α degradation, reducing hypoxia-driven tumor growth 15.

Beyond proliferation, ERβ also inhibits cell migration and epithelial-mesenchymal transition (EMT). Suppressed ERβ expression, due to factors like TGFβ or hypoxia, enhances EMT in PCa cells 10. Restoring or activating ERβ increases apoptosis and reduces migration, highlighting its role in metastasis inhibition. ERβ activation induces FOXO3a expression, which upregulates the pro-apoptotic factor PUMA, triggering apoptosis through p53-dependent and -independent pathways 16. Adenoviral restoration of ERβ in DU145 cells inhibits proliferation and invasion 17. ERβ also regulates metabolic, survival, and antioxidant pathways (e.g., PI3K, Akt), reducing oxidative stress and reinforcing its potential as a therapeutic target 18.

Several isoforms of ERβ (e.g., ERβ1, ERβ2, ERβ5) are expressed in prostate cells, with ERβ1 being the most active isoform in transcription. ERβ2 and ERβ5 have reduced activity and are linked to increased tumor invasiveness, suggesting that isoform expression modulates PCa aggressiveness 19,20. ERβ2 stabilizes HIF-1α under normoxic conditions, inducing hypoxia-related genes that drive metastasis and angiogenesis 21. These findings underscore the role of ERβ isoforms in PCa progression and chemotherapy resistance. ERβ2 and ERβ5 contribute to stem-like traits and reduced chemoresponsiveness, while ERβ1 generally enhances chemosensitivity.

##### 2.2.1.2 ERα in Prostate Cancer

ERα, discovered by Elwood Jensen over 60 years ago and cloned in 1985 22, is predominantly expressed in stromal cells of the prostate, with minimal expression in epithelial cells 23. Knockout mouse models have shown that estrogens promote prostate cancer (PCa) progression via ERα 24. Its expression is elevated in higher-grade prostate cancers, linking it to tumor progression 25. ERα's oncogenic functions are mediated through signaling pathways like MAPK/ERK, PI3K/AKT, and β-catenin 1. Recent studies identified ERα mutations and isoforms, including ERα-36, which activates nongenomic signaling pathways, enhancing metastatic potential and promoting resistance to anti-estrogen therapies 26. ERα-36 also mediates estrogen-induced epithelial-mesenchymal transition, osteoclastogenesis, and lung metastasis, making it a potential therapeutic target in metastatic PCa 27.

The estrogen signaling pathway in PCa is complex, with ERα typically exhibiting oncogenic characteristics by promoting cell proliferation, migration, and resistance mechanisms. In contrast, ERβ, particularly ERβ1, acts as a tumor suppressor by inhibiting the cell cycle, inducing apoptosis, and regulating key molecules like PTEN and HIF-1α. However, ERβ isoforms like ERβ2 and ERβ5 may enhance invasiveness and stemness, reducing chemotherapy response 28. ERα and ERβ are not always antagonistic; in some PCa cell lines, both receptors cooperate to activate the SRC and PI3K/AKT pathways, promoting migration, invasion, and colony formation 29. The interplay between ERα and ERβ can be influenced by their co-expression and signaling crosstalk in specific cellular environments.

##### 2.2.1.3 GPER in Prostate Cancer

GPER, a membrane-bound receptor, adds to the complexity of estrogen signaling in PCa. Its activation can inhibit tumor growth by inducing cell cycle arrest, but in cooperation with ERα or ERβ, it may promote proliferation and metastasis. Additionally, estrogen signaling, particularly through ERβ, inhibits androgen signaling, suggesting a key role in PCa progression.

In conclusion, the estrogen pathway in PCa is a multilayered system shaped by receptor subtype combinations, signaling crosstalk, and external stimuli. This complexity underscores the need for individualized clinical strategies based on receptor expression profiles and signaling states for precise intervention in PCa treatment.

#### 2.2.2 Inhibition or Synergistic Effects of Estrogen on Androgen Signaling

Androgen receptor (AR) signaling is essential for prostate cancer initiation and progression. ER and AR signaling interact in PCa, with CAMKK2, a downstream AR target, influencing cell survival, metabolism, and migration 30. ERβ activation suppresses AR expression and its downstream activity, suggesting ERβ as a negative regulator of AR signaling in PCa. Targeting ERβ may offer an alternative strategy to bypass AR-driven resistance, as activating ERβ with specific agonists like 8β-VE2 reduces cell survival and increases apoptosis in AR-positive PCa cells during androgen deprivation 31.

PCa is often treated with androgen deprivation therapy (ADT), but resistance often leads to CRPC. ERβ activation can help overcome ADT resistance, potentially offering a novel treatment strategy. GPER, located in the plasma membrane and endoplasmic reticulum, also modulates androgen signaling. Activation of GPER leads to G2-phase cell cycle arrest in PCa cells, suppressing growth 32. Interestingly, GPER's effects are context-dependent, and in combination with ERα and ERβ, it can promote proliferation and metastasis. GPER activation induces cell cycle arrest and suppresses migration and invasion through p21 expression 33.

GPER's role is further modulated by ERα and ERβ, as their activity can disrupt GPER-mediated signaling, shifting it toward tumor progression. ERα and ERβ synergistically regulate key genes (e.g., c-Fos, CTGF, EGR1) involved in cell proliferation and survival, contributing to PCa progression 34. Thus, GPER's dual role in PCa makes it a promising therapeutic target, with the potential to be modulated depending on the tumor's receptor profile.

#### 2.2.3 Estrogen Receptor Signaling and Its Association with Prostate Cancer Risk and Prognosis

Genetic variations in estrogen receptors (ERα and ERβ) are linked to prostate cancer risk and prognosis. For instance, ESR1 rs9340799 (XbaI) polymorphism significantly increases PCa risk, especially in Caucasian populations 35. The ESR2 rs1256049 variant has ethnic differences, increasing risk in Caucasians but offering a protective effect in Asians 36. These findings suggest that ER polymorphisms can predict PCa susceptibility and may be involved in the molecular pathogenesis of PCa.

The expression of ER in PCa tissues is correlated with treatment efficacy. ER-negative or low-expressing tumors show a poorer response to endocrine therapies, highlighting ER status as a potential biomarker for therapeutic outcomes. Selective estrogen receptor modulators (SERMs) like toremifene reduce vertebral fracture incidence and improve bone density in PCa patients on ADT, suggesting their role as adjunctive therapies 37. This supports the potential use of ER-targeting agents in improving patient outcomes during ADT.

In CRPC, overexpression of ESR1 represents a unique molecular subtype with a poorer prognosis. This subtype activates key oncogenic pathways, such as PI3K/Akt and AR signaling, making it a target for ERα-based therapies. Tamoxifen and other ER-targeting drugs like ISA-2011B may improve treatment responses in ESR1-positive, castration-resistant patients 38.

In conclusion, estrogen receptor signaling is critical not only in genetic susceptibility to prostate cancer but also in its progression and treatment response. Combining ER gene polymorphisms with tissue ER expression status can improve risk stratification and guide personalized treatment strategies. Targeting ER pathways in combination with existing therapies offers an opportunity for more precise interventions in prostate cancer.

### 2.3 Estrogen and Bladder Cancer

#### 2.3.1 Regulatory Role of ERα and ERβ in Bladder Cancer

Bladder cancer is the fourth most common malignancy worldwide, with significant mortality rates. Recent studies indicate that estrogen receptors play a crucial role in the initiation and progression of bladder cancer, particularly in non-muscle-invasive bladder cancer (NMIBC).

##### 2.3.1.1 ERα's Role in Bladder Cancer

ERα expression in bladder cancer tissues is generally low, with higher expression of ERβ observed in both epithelial and stromal cells, particularly in high-grade and muscle-invasive bladder cancer (MIBC) 2,39. ERα is more commonly found in NMIBC, but its expression decreases as tumors progress to high-grade and metastatic stages 2. In contrast, ERβ is more strongly associated with higher tumor grades and stages, and its positive expression may serve as an independent predictor of disease progression in NMIBC 40. These findings suggest distinct roles for ERα and ERβ in bladder cancer progression, with ERβ considered the predominantly expressed estrogen receptor in urothelial carcinoma 2,40.

ERα regulate bladder cancer cell proliferation by binding to estrogen or selective estrogen receptor modulators. Studies show that ERα activation promotes cell cycle progression, accelerating proliferation by increasing cyclins D1 and E 41. ERα is also expressed in the stroma, where it enhances tumor invasiveness through pathways involving CCL1 and IL-6^42^. Interestingly, ERα's activation in urothelial cells can protect against malignant transformation in some models 43. Additionally, ERα inhibits AKT signaling through INPP4B induction, suppressing bladder cancer cell growth 43.

##### 2.3.1.2 ERβ's Role in Bladder Cancer

On the other hand, ERβ activation promotes bladder cancer progression by modulating pathways like MCM5, CCL2, and IL-1/c-Met 44-46. ERβ also influences the NF-κB pathway, a key regulator of inflammation, which suggests its role in malignant transformation 47. Interestingly, ERβ may have a tumor-suppressive effect in some contexts, such as inhibiting migration and invasion through upregulation of E-cadherin and downregulation of N-cadherin 47,48. In contrast, ERβ activation in certain models promotes tumor cell proliferation, suggesting a dual role depending on tumor context and microenvironment. Additionally, ERβ suppression of MCM5 expression highlights its involvement in regulating DNA replication and inhibiting tumor cell proliferation 45.

ER signaling also interacts with noncoding RNAs in bladder cancer. For instance, ERα induces miR-4324 expression, inhibiting cell proliferation and migration 49. Conversely, ERβ upregulates miR-92a, promoting proliferation and invasion 50. Estrogen-responsive eRNAs like eGREB1 and P2RY2e also play significant roles in tumor cell behaviors 51,52. These findings indicate that estrogen receptor signaling and noncoding RNA regulation are closely intertwined in bladder cancer progression, offering potential therapeutic targets for the disease.

#### 2.3.2 Gender Differences and the Impact of Estrogen on Bladder Cancer Progression

Epidemiological studies show that bladder cancer incidence is higher in men, but women experience more aggressive tumors and worse prognosis 53,54. Despite this, bladder cancer in women is often misdiagnosed due to overlapping symptoms with urinary tract infections, leading to delayed treatment 55. Sex differences in bladder cancer progression are likely linked to estrogen receptor signaling, with female sex serving as an independent factor for poor prognosis and response to therapy 56.

Estrogen receptors' expression levels are closely associated with bladder cancer progression. Low expression of UGT1A, a detoxifying enzyme, is linked to high-grade tumors and poor survival in MIBC 57. Estrogen stimulation upregulates UGT1A in normal urothelium, but decreases its expression in tumor cells, suggesting estrogen's role in regulating carcinogen detoxification 58. Additionally, androgen signaling may suppress UGT1A, interacting with estrogen pathways to modulate bladder cancer progression 59.

Moreover, enzymes involved in estrogen metabolism, such as aromatase and steroid sulfatase, are downregulated in advanced bladder cancers, potentially contributing to increased tumor progression due to lower bioavailable estrogen levels 60. Higher levels of estrogen sulfotransferase (EST) and aromatase are associated with higher tumor grade, indicating that localized estrogen production may be linked to more aggressive disease 60.

#### 2.3.3 Estrogen Receptor Signaling and Its Association with Bladder Cancer Risk and Prognostic Outcomes

ERβ expression correlates with higher-grade and MIBC, and its positive expression is an independent risk factor for recurrence and progression in NMIBC 61. Studies show that ERβ-positive tumors have poorer recurrence-free survival (RFS) and are more likely to invade muscle tissue 40. Aromatase expression, which often co-exists with ERβ in invasive tumors, is associated with advanced tumor stages, positive lymph node status, and increased cancer-specific mortality 62. ERβ activation enhances bladder cancer cell proliferation and could contribute to resistance to certain therapies 60.

In contrast, ERα expression is lower in bladder cancer, and its expression increases in metastatic tumors. In NMIBC, ERα positivity may be associated with a lower recurrence risk (P = 0.0746) 2. This suggests ERα might play a tumor-suppressive role in early-stage bladder cancer.

In conclusion, estrogen receptor signaling plays a dual role in bladder cancer. High ERβ expression is typically associated with malignancy, poor prognosis, and resistance to treatment, while ERα shows stage-dependent effects: it may act as a tumor suppressor in early-stage bladder cancer but could have oncogenic effects in advanced stages. The interaction between ERα and ERβ and their differential roles in various contexts underscore the complexity of estrogen signaling in bladder cancer. Targeting estrogen receptor pathways may offer new opportunities for therapy, particularly for patients with high ERβ expression.

### 2.4 Estrogen and Kidney Cancer

#### 2.4.1 Function and Expression of Estrogen Receptors in Renal Cell Carcinoma

Renal cell carcinoma (RCC) is the most common kidney cancer, with clear cell renal cell carcinoma (ccRCC) being the most prevalent subtype. Estrogen receptors, including the nuclear ERα and ERβ, and the membrane receptor GPER, are expressed in both normal renal tissues and RCC, but their expression levels vary significantly across RCC subtypes. In particular, ERβ expression is notably higher than ERα in RCC, with some studies reporting a complete absence of ERα in certain RCC cell lines and tissues 3,63,64.

##### 2.4.1.1 ERα in Renal Cell Carcinoma

ERα is involved in regulating key DNA repair mechanisms and genomic stability. It modulates the DNA damage response (DDR) by interacting with DNA repair proteins like FEN1, APE1, Ku70, and others 65. In specific RCC subtypes like Xp11.2 translocation RCC, ERα plays a pivotal role in tumor progression through estrogen-dependent pathways 65. Additionally, the ERα variant ERα36 is linked to poor prognosis and metastasis 66.

The von Hippel-Lindau (VHL) gene mutation, common in ccRCC, leads to increased ERα expression, which enhances HIF-1α transcription, further promoting tumor growth 67,68. On the other hand, ERβ shows both tumor-suppressive and tumor-promoting effects, depending on its expression and signaling pathways 69,70. ERβ activation in RCC cell lines inhibits proliferation and migration, while its overexpression can contribute to the cancer stem cell phenotype by regulating specific miRNAs 71.

##### 2.4.1.2 ERβ and GPER in Renal Cell Carcinoma

ERβ also enhances tumor cell invasiveness by modulating pathways such as TGF-β1/SMAD3 and VEGFa/HIF2α 72,73. It promotes angiogenesis in RCC through the LncRNA-SERβ/ERβ/ZEB1 axis 3. However, in some cases, ERβ activation may also increase resistance to treatments, including TKIs 74. GPER, another estrogen receptor, has been found to influence RCC progression through the PI3K/AKT/MMP signaling pathway 75. Interestingly, agonists like G-1 may help overcome resistance to sunitinib by modulating phosphorylation pathways 75,76.

In conclusion, estrogen signaling in RCC is complex, with ERα and ERβ having opposing roles depending on the tumor subtype and microenvironment. While ERα may contribute to tumor progression, especially in specific subtypes like Xp11.2 translocation RCC, ERβ may exert tumor-suppressive effects in early stages, but shift to a tumor-promoting role in more advanced stages 77. Understanding these mechanisms offers new therapeutic opportunities, particularly in targeted and personalized RCC treatments.

#### 2.4.2 Association Between Estrogen and Gender Differences in Kidney Cancer

Epidemiological data show a higher RCC incidence in men, with a 2:1 male-to-female ratio. Men also tend to have larger tumors and more advanced disease stages. Notably, women are underrepresented in papillary RCC but overrepresented in chromophobe RCC 78. Interestingly, young women with RCC have lower cancer-specific mortality rates compared to men, although this gap narrows post-menopause 79.

These sex differences may be partially attributed to estrogen signaling, which has been shown to reduce proliferation and migration in RCC cell lines, particularly those with high ERβ expression 70. Estrogen may help clear cancer cells by activating ERβ's tumor-suppressive functions. Additionally, the higher incidence of Xp11.2 tRCC in females further supports estrogen's role in RCC sex differences 65.

X-chromosome genes like KDM5C and KDM6A also contribute to these differences. KDM5C mutations are more common in male RCC patients, which may explain the higher RCC incidence in men 80. Furthermore, sex-specific immune responses in the tumor microenvironment (TME) suggest that estrogen's immunomodulatory effects could influence RCC progression and treatment responses 81.

In conclusion, RCC exhibits significant sex-based differences in incidence, progression, and treatment response. These findings emphasize the need for sex-specific therapeutic strategies to improve treatment efficacy for both men and women with RCC.

#### 2.4.3 Estrogen Receptor Signaling and Its Association with Prognosis and Treatment Response in RCC

ERα36 expression is linked to poor prognosis in RCC, with high levels correlating with reduced disease-free survival (DFS) and overall survival (OS). Membrane-localized ERα36 is especially indicative of malignant tumors, while its absence is more common in benign ones 66.

A study of advanced RCC patients showed that tamoxifen treatment led to disease stabilization in some patients, particularly those in good physical condition. Although the response rate to tamoxifen was low, these findings suggest a potential for estrogen signaling intervention in combination therapies 82.

In conclusion, estrogen receptor signaling is integral to RCC progression and prognosis. While ERα36 can serve as a poor prognostic marker, targeting estrogen receptors may help delay disease progression, especially when combined with other therapeutic strategies 66,82.

## 3. Applications of Estrogen-Targeted Therapy in Urological Tumors

### 3.1 Estrogen-Targeted Therapy in Prostate Cancer

#### 3.1.1 Estrogen Receptor Inhibitors and Treatment Strategies for Prostate Cancer

In PCa treatment research, selective activation of estrogen receptor subtypes, particularly ERβ, has demonstrated significant anti-cancer potential. Compounds such as raloxifene, tamoxifen, genistein, and curcumin selectively activate ERβ, effectively inhibiting prostate cancer cell proliferation and migration 83. These effects are primarily achieved by modulating pathways related to cell cycle regulation and cholesterol biosynthesis, thereby suppressing cancer cell activity. Furthermore, research indicates that non-estrogen ligands, such as Fulvestrant (ICI 182,780) and flavonoid compounds, can also exert anticancer effects via the ERβ pathway, independent of the classical estrogen response elements. Their anticancer activity is mainly achieved by modulating signaling pathways such as NF-κB or Sp1, which inhibit PCa cell proliferation.

Tamoxifen has shown remarkable efficacy against CRPC, demonstrating inhibitory effects in both *in vitro* experiments and *in vivo* models. When combined with ISA-2011B, a PIP5K1α inhibitor, tamoxifen's anti-cancer activity is further enhanced. This combination therapy more effectively suppresses CRPC cell proliferation, suggesting its potential utility in treating resistant forms of prostate cancer. It is also noteworthy that the GPR30 agonist G-1 can inhibit PCa cell growth by activating the Erk1/2 signaling pathway, significantly reducing cell migration and invasion. This suggests that GPR30 may serve as another critical therapeutic target 84.

Raloxifene, a selective estrogen receptor modulator, exhibits multi-layered anti-PCa activity. Studies reveal that raloxifene can activate various signaling pathways to induce cell death and inhibit proliferation in prostate cancer cells with differing ERα and ERβ expression levels 85. Additionally, raloxifene downregulates GPR30/GPER1 signaling, significantly reducing the viability and migratory capacity of LNCaP cells 86. Importantly, raloxifene induces apoptosis in androgen-responsive prostate cancer cell lines such as LNCaP, independent of androgen signaling. This dual function as an estrogen receptor agonist/antagonist underscores raloxifene's ability to control the progression of androgen-independent prostate cancer.

SERMs and selective estrogen receptor degraders (SERDs) also show promise in immunotherapy. Tamoxifen and fulvestrant not only effectively inhibit cancer cell proliferation but also enhance the activity of natural killer (NK) cells without damaging healthy tissues. By increasing NK cell cytotoxicity and promoting immunological synapse formation, these agents facilitate the lysis of tumor cells. Furthermore, fulvestrant reduces immunosuppressive myeloid-derived suppressor cells (MDSCs) and regulatory T cells (Tregs) in xenograft tumor models while increasing infiltration of dendritic cells (DCs), CD8+ T cells, and CD4+ T cells. This immune-modulatory effect significantly enhances the efficacy of PD-L1 immune checkpoint inhibitors 87.

Interestingly, fulvestrant has been found to enhance the sensitivity of enzalutamide-resistant prostate cancer cells to NK cell-mediated cytotoxicity. This finding provides theoretical support for combining fulvestrant with NK cell-based immunotherapy, offering a novel approach to overcoming treatment resistance in clinical settings.

#### 3.1.2 Potential Applications of Combination Therapy with Androgen Blockade

Combined androgen blockade (CAB) demonstrates significant potential in PCa treatment. Bicalutamide, a nonsteroidal anti-androgen, is a first-line therapy for advanced PCa. However, patients often develop resistance to bicalutamide shortly after initiation, limiting its therapeutic efficacy. Research has identified activation of the ERα-NRF2 (nuclear factor erythroid 2-related factor 2) signaling pathway as a key mechanism underlying bicalutamide resistance 88. Further studies reveal that tamoxifen can effectively suppress this pathway, reversing resistance to bicalutamide in resistant cells. This finding supports the development of combination therapies using bicalutamide and tamoxifen as a novel treatment strategy.

In preclinical trials, fulvestrant has exhibited anti-proliferative activity in both androgen-sensitive and androgen-resistant prostate cancer cells, particularly in the LNCaP cell line. Fulvestrant significantly inhibits the growth of these cells by downregulating AR expression levels 89. It not only reduces AR mRNA and protein levels but also decreases the dependence of prostate cancer cells on androgens, providing a new approach for addressing bicalutamide resistance.

Further research indicates that enhanced EGFR and ERβ signaling pathways may play crucial roles in the progression of prostate cancer from localized lesions to metastatic disease. Consequently, combination therapies targeting these pathways are gaining attention. For instance, a therapeutic regimen combining tamoxifen, gefitinib, and etoposide has demonstrated superior anti-tumor effects across multiple prostate cancer cell lines. Compared to monotherapies, dual or triple combinations significantly inhibit cancer cell proliferation and induce apoptosis, thereby improving therapeutic outcomes 90.

#### 3.1.3 Estrogen Receptor-Targeted Therapy in Prostate Cancer: Clinical Studies

Currently, research on estrogen receptor-related drugs in PCa primarily falls into three categories: plant-derived estrogen analogs, SERMs, and estrogen receptor antagonists or degraders.

##### 3.1.3.1 Plant-Derived Estrogen Analogs

Plant-based estrogen analogs such as daidzein have shown certain biological effects in patients with localized prostate cancer. A Phase II double-blind, placebo-controlled trial demonstrated that prostate cancer patients treated with 30 mg/day of daidzein for 3-6 weeks prior to surgery had a 7.8% decrease in PSA levels, while the placebo group showed a 4.4% increase (P = 0.051). Furthermore, this compound significantly reduced serum total cholesterol levels (P = 0.013) without affecting sex hormones, and was well tolerated 91. Another Swedish case-control study involving 1314 prostate cancer patients and 782 controls found an interaction between plant estrogen intake and ERβ promoter polymorphism (rs2987983). In individuals carrying the variant allele, high plant estrogen intake significantly reduced prostate cancer risk (odds ratio of the highest vs. lowest quartile = 0.43, P < 0.001), suggesting that plant estrogens may have population-specific preventive effects 92.

##### 3.1.3.2 SERMs

SERM drugs, including toremifene, tamoxifen, and raloxifene, have demonstrated anticancer potential in both clinical and animal studies. Toremifene has yielded positive results in multiple clinical studies. In a Phase IA trial, 21 patients with high-grade prostatic intraepithelial neoplasia (PIN) showed 72% of cases with no residual PIN in biopsies after treatment, compared to 17.9% in historical controls 93. In a Phase IIB double-blind, placebo-controlled study with 514 participants, the annual incidence of prostate cancer was 24.4% in the 20 mg toremifene group, significantly lower than the 31.2% in the placebo group (P < 0.05). In those without cancer at 6 and 12 months, the incidence dropped further to 9.1% (compared to 17.4% in the placebo group) 94. Animal models also support its efficacy: in TRAMP mice, toremifene delayed tumor formation to 29 weeks (compared to 17 weeks in the control group), and only 35% of toremifene-treated animals developed tumors by week 33, whereas 100% of control animals did 95. Raloxifene has also been shown to induce apoptosis and inhibit proliferation via ERα/β signaling. In contrast, tamoxifen demonstrated limited clinical activity in a Phase II trial involving 30 patients with hormone-refractory prostate cancer at a high dose of 160 mg/m²/day, with only one case of partial response (3.3%) and six cases of stable disease (20%), yielding an overall response rate of 23%, and a median survival of 10.5 months 10. It suggests limited clinical efficacy, but some biological activity. Notably, the novel SERM, omerosifene, has shown the potential to inhibit both androgen-dependent and independent prostate cancer cell growth *in vitro* and animal models, affecting multiple oncogenic signaling pathways. However, it lacks clinical data and requires further investigation 96.

##### 3.1.3.3 SERDs

Regarding ER antagonists/degraders, fulvestrant, a pure ER antagonist, failed to produce significant PSA responses in a Phase II clinical trial involving 20 patients with CRPC, with no patient showing more than a 50% reduction in PSA. The median progression-free survival was only 4.3 months, and the median overall survival was 19.4 months 97. Conversely, a small retrospective study showed that after high-dose fulvestrant, some patients had a PSA reduction of up to 68.3%, suggesting a potential dose-dependent response, though overall evidence remains inconsistent 98. Additionally, early use of synthetic estrogens like diethylstilbestrol can effectively inhibit tumors, but high doses are associated with severe cardiovascular adverse events, limiting their widespread use.

In conclusion, estrogen receptor-related drugs show promising clinical potential in prostate cancer, particularly SERMs, which have demonstrated preventive potential in high-risk populations. However, current studies still face challenges such as small sample sizes, limited efficacy, or inconsistent data. Future research should further clarify the mechanisms of action, optimize dosing regimens, and explore precision treatment strategies that match individual genotypes, thereby advancing their clinical translation in prostate cancer treatment and prevention.

#### 3.1.4 Prospects of Estrogen Receptor-Targeted Therapy in Castration-Resistant Prostate Cancer

Estrogen-targeted therapy is gaining increasing attention in the treatment of CRPC, particularly for ER-positive metastatic CRPC subtypes. Research has shown that tamoxifen offers a novel therapeutic approach by targeting ERα. The anti-tumor mechanisms of tamoxifen primarily involve inhibition of signaling pathways such as PI4P5K-α/AKT and MMP-9/VEGF, which suppress cancer cell proliferation and metastasis 87. These findings suggest that tamoxifen may have therapeutic value in ER-positive CRPC patients. However, given the anti-proliferative effects of ERβ and its reduced expression in high-grade tumors, a single-agent ER-targeted therapy may be insufficient to fully control cancer progression. Therefore, future treatment strategies may require combination therapies to enhance efficacy.

Raloxifene is another SERM with promising potential. In a phase II clinical trial, raloxifene significantly inhibited tumor growth and delayed disease progression in CRPC xenograft models. Additionally, in CRPC orthotopic models, raloxifene reduced tumor volume by 70% and lymph node size by 62%, with no observed lung metastases. These effects are likely associated with its ability to substantially reduce ERα and ERβ expression levels by 84% and 92%, respectively, demonstrating its tumor-suppressive and anti-metastatic potential 99.

Fulvestrant, an estrogen receptor degrader, also shows dose-dependent efficacy in CRPC. In one study, among seven heavily pretreated CRPC patients, six showed significant declines in PSA levels following an initial dose of fulvestrant at 500 mg. However, when the dose was reduced to 250 mg, PSA levels rebounded, highlighting the therapy's sensitivity to dose adjustments 98. This finding underscores the importance of determining the optimal dosing regimen for fulvestrant to maximize its therapeutic efficacy.

In summary, estrogen-targeted therapy exhibits promising clinical potential in CRPC treatment. However, due to the complex roles of ER receptors, relying solely on single-agent ER-targeted drugs may not suffice to completely inhibit tumor progression. Future research should focus on exploring multi-targeted combination therapies and optimizing dosing strategies to improve therapeutic outcomes and patient prognosis.

### 3.2 Estrogen-Targeted Therapy in Bladder Cancer

#### 3.2.1 Research on Estrogen Receptor Antagonists in Bladder Cancer Treatment

SERM have garnered significant attention in recent bladder cancer research. These compounds have been repeatedly reported to inhibit the proliferation and invasion of bladder urothelial carcinoma cells 100. SERMs exhibit tissue-selective actions, acting as ER agonists in tissues like bone, liver, and the cardiovascular system, while functioning as ER antagonists in the breast and uterus 101.

Estrogen plays a crucial role in the onset and progression of bladder cancer, particularly in ER-positive patients. Studies have shown that E2 (estradiol) significantly promotes the proliferation of bladder cancer cells, such as HTB-1, HTB-3, and HTB-5. SERMs, as well as ERβ antagonists like tamoxifen, can effectively inhibit the proliferation of HTB-1 and HT1376 cells 39. Research by Kim *et al.* further demonstrated that raloxifene could induce apoptosis in UCB (urothelial carcinoma of the bladder) cell lines 102. In addition, Sonpavde *et al.* found that tamoxifen and raloxifene significantly inhibited the growth of 5637 cell xenograft tumors in nude mice 100. Despite the proven efficacy of SERMs in certain UCB cell lines and mouse models, the issue of targeting ER subtypes remains unresolved 66. Studies have indicated that the roles of ERα and ERβ in bladder cancer might be opposite, which is related to the expression distribution of their different subtypes and variants. Further research revealed that ERβ can enhance bladder cancer cell proliferation and invasion by regulating the miR-92a/DAB2IP (DOC-2/DAB2 Interaction Protein) signaling pathway 50. Small molecule drugs targeting the ERβ/miR-92a/DAB2IP signaling axis have shown significant antitumor effects in animal models. Moreover, estrogen signaling is closely linked to chemotherapy resistance in bladder cancer 61. E2 inhibits the adhesion and internalization of BCG (Bacillus Calmette-Guérin) and suppresses immune cell recruitment via ER, thereby weakening the efficacy of BCG therapy. However, tamoxifen and ICI 182,780 (Fulvestrant) can reverse this effect 105. Additionally, ERα can enhance the cytotoxicity of doxorubicin by inducing the expression of miR-4324^49^, thereby improving BCG efficacy. In contrast, ERβ signaling may promote resistance by inactivating FOXO1. These findings suggest that targeting ER subtypes or combining anti-estrogen drugs with BCG or chemotherapy agents holds promise in overcoming resistance and delaying tumor progression.

In bladder cancer studies, SERM drugs such as tamoxifen, raloxifene, and PHTPP have demonstrated efficacy in suppressing bladder cancer cell growth. Specifically, in carcinogen-induced bladder cancer models, treatment with the selective ERβ antagonist PHTPP showed effects similar to ERβ knockout, significantly reducing bladder cancer incidence in female mice. Following SERM treatment, the average tumor volume in bladder cancer-bearing nude mice decreased, with 17 of 30 treated mice showing no detectable tumors 100. Moreover, the tumor incidence in treated mice dropped from 76% in the control group (BBN alone) to 10-14% 106.

In preclinical models, E2 promoted the proliferation of ERα-positive bladder cancer cell lines, whereas SERMs (including tamoxifen and raloxifene) and the pure anti-estrogen ICI 182,780 inhibited cell growth 47,61,107,108. Notably, SERMs also demonstrated significant inhibitory effects in ERα-negative/ERβ-positive bladder cancer cells, while their impact on ERα/ERβ double-knockdown cell lines was limited. These findings suggest that ER signaling generally promotes bladder urothelial carcinoma progression, and intervention with selective ER modulators offers a potential therapeutic strategy.

In addition to estrogen and its receptors, recent research has started to focus on the therapeutic potential of related metabolic enzymes, such as aromatase and steroid sulfatase (STS). The expression of these two enzymes in bladder urothelial carcinoma has been found to be negatively correlated with tumor progression 60, suggesting that a reduction in estrogen levels may accelerate tumor deterioration, leading to poorer prognosis. Given the success of aromatase inhibitors in the treatment of postmenopausal breast cancer 101, the introduction of these inhibitors into bladder cancer therapy has been proposed as a new strategy.

#### 3.2.2 Combination of Estrogen-Targeted Therapy and Immunotherapy in Bladder Cancer

ER modulators not only exert direct inhibitory effects on the initiation and progression of BCa but can also enhance therapeutic outcomes when combined with existing treatments such as chemotherapy and immunotherapy. Currently, cisplatin-based combination chemotherapy regimens, such as MVAC (methotrexate/vinblastine/doxorubicin/cisplatin) and GC (gemcitabine/cisplatin), remain the primary therapeutic approaches for locally advanced or metastatic BCa. However, complete eradication is often challenging due to the development of drug resistance. Studies have indicated that ER signaling may influence BCa chemosensitivity. In a preclinical study, tamoxifen, used as a chemosensitizer, enhanced the response of bladder cancer cells to methotrexate, vinblastine, doxorubicin, and cisplatin in a concentration-dependent manner 109.

In the context of Bacillus Calmette-Guérin (BCG) immunotherapy, ER signaling also demonstrates regulatory potential. Although data indicate the presence of gender-related differences, both male and female BCa patients generally exhibit low sensitivity to BCG treatment, suggesting that sex hormone receptor signaling may impact BCG efficacy. In ERα-positive/ERβ-positive bladder cancer cells, E2 reduced BCG attachment, internalization, and the recruitment of monocytes/macrophages. Conversely, tamoxifen and the pure anti-estrogen ICI 182,780 reversed the inhibitory effects of E2 and enhanced BCG-induced cytotoxicity in bladder cancer cells and mouse models 105. *In vivo* studies further revealed that the combination of ICI 182,780 with BCG was more effective in suppressing BCa growth compared to BCG monotherapy. Mechanistic investigations suggest that ICI 182,780 enhances tumor suppression induced by BCG by increasing integrin-α5β1 expression and interleukin-6 (IL-6) release, thereby facilitating BCG attachment and internalization in bladder cancer cells 105.

Additionally, ER activation may modulate the response of bladder cancer to immune checkpoint inhibitors, such as PD-1/PD-L1 inhibitors. Similar to observations in breast cancer 110, ER activation may reduce sensitivity to PD-1/PD-L1 inhibitors. Therefore, exploring the role of ER signaling in the immunotherapy of bladder cancer is of significant interest. In summary, ER modulators hold great potential in the treatment of BCa, especially in combination with existing chemotherapy and immunotherapy, offering prospects for improved therapeutic outcomes.

#### 3.2.3 Estrogen-Targeted Therapy Strategies Supported by Clinical Data and Experimental Research

The clinical application of SERMs has made significant progress, particularly in endocrine therapy for breast cancer, where their role has markedly reduced recurrence and mortality rates. Similarly, research into the application of SERMs in BCa has shown promising potential. In recent years, an increasing number of studies have explored the potential of ER-targeted therapies in BCa, focusing on improving treatment efficacy by modulating ER signaling. Currently, two phase II clinical trials related to ER signaling in BCa are underway. One trial (NCT02197897) aims to evaluate the efficacy of tamoxifen in patients with NMIBC, with a primary focus on clinical responses after 12 weeks of treatment and immunohistochemical changes in biomarkers such as ERα and ERβ in post-treatment biopsy specimens 111. Another trial (NCT01489813) investigates the potential therapeutic effects of the phytoestrogen genistein 112. This study not only examines genistein's ability to mitigate adverse urinary symptoms associated with intravesical BCG therapy but also explores whether it can enhance the antitumor effects of BCG. In this trial, patients with superficial BCa received oral genistein for 10 weeks during and after BCG therapy, and outcomes were compared to a placebo group to evaluate changes in urinary symptoms and tumor recurrence rates.

Although these trials demonstrate the potential application of ER-targeted therapies in BCa, their clinical benefits require further validation. Preclinical data suggest that anti-estrogen therapies might enhance sensitivity to conventional non-surgical treatments for BCa. However, these findings must be substantiated through larger-scale clinical studies. Furthermore, it is essential to explore the expression of ERα and ERβ in clinical specimens and their role as potential biomarkers to more accurately predict treatment responses. Such efforts could provide insights into ER functional activity in surgical specimens and help forecast patient prognosis, thereby informing future therapeutic strategies.

Tamoxifen has also garnered attention as a potential therapeutic agent in advanced BCa. Early case reports indicate its therapeutic effects in metastatic urothelial carcinoma. For example, one male patient experienced unexpected regression of metastatic cancer following tamoxifen treatment for gynecomastia 113. Additionally, in a study investigating tamoxifen's chemosensitizing effects, 30 BCa patients—including those with muscle-invasive and metastatic BCa—were treated with standard chemotherapy (cisplatin, methotrexate, and vinblastine [CMV]) combined with high-dose tamoxifen. Although the lack of a control group limits the study's conclusions, the observed overall response rate of 58% was comparable to the efficacy of traditional chemotherapy regimens 114.

In summary, existing clinical and experimental data preliminarily support the potential application of ER-targeted therapies in BCa. However, further randomized controlled trials are necessary to clarify the clinical benefits of anti-estrogen or estrogen-based therapies for BCa patients and to establish the role of ER expression in guiding treatment decisions. Such efforts are critical to advancing the clinical translation of ER-targeted therapies in the field of BCa.

### 3.3 Estrogen-Targeted Therapy in Kidney Cancer

#### 3.3.1 Combining Estrogen Receptor-Targeted Therapy with Existing Kidney Cancer Treatments

In the past decade, significant advancements have been made in the treatment of RCC. Current non-surgical approaches include tyrosine kinase inhibitors (TKIs) such as sorafenib and sunitinib, monoclonal antibodies like bevacizumab, and immunotherapies targeting PD-1/PD-L1 inhibitors. However, curative treatment remains challenging, especially for advanced RCC. In recent years, combination therapies for RCC have demonstrated superior efficacy, with several combinations now recommended as first-line treatments, positioning them as promising strategies for RCC management 115.

It is noteworthy that the five-year survival rate for metastatic RCC remains below 20%, highlighting the urgent need for the exploration of new targets and therapies 116. Current clinical treatments mainly rely on VEGF pathway inhibitors (e.g., sunitinib), mTOR inhibitors (e.g., everolimus), and other targeted therapies 117. However, their effectiveness is limited by the development of resistance and the differential response of various metastatic sites to treatment, especially in non-clear cell carcinoma where treatment efficacy is relatively poor. Consequently, studies have found that ERβ expression is elevated in high-grade RCC, making it a potential prognostic biomarker and therapeutic target 72. In animal models, anti-estrogen drugs such as nafoxidine and the ERβ selective antagonist PHTPP have been shown to significantly inhibit RCC growth and invasion 72, suggesting that targeting the ERβ signaling pathway could be an effective strategy for developing novel treatment methods.

Research into ER in RCC suggests that ER-targeted approaches hold significant potential in combination therapies. A study involving 10 patients with advanced RCC employed a combined chemoendocrine treatment regimen that included tegafur, a fluorouracil prodrug, and tamoxifen. Positive responses were observed in both ER-positive and ER-negative tumor subtypes 118, suggesting that the ER pathway might play a role across various RCC subtypes.

Sunitinib, a first-line TKI for RCC, has been reported to increase cancer stem cells and promote vasculogenic mimicry through the regulation of the lncRNA-ECVSR/ERβ/HIF2α signaling axis 119, contributing to treatment insensitivity and resistance. Mouse model studies demonstrated that combining the small-molecule anti-estrogen PHTPP with sunitinib significantly enhances sunitinib's efficacy. Furthermore, another study revealed that the FDA-approved anti-estrogen drug fulvestrant targets ERβ and improves sunitinib sensitivity in RCC by modulating the ERβ/ANGPT-2/Tie-2 signaling pathway 74. These findings underscore the potential for developing new combination therapies with sunitinib. The G protein-coupled estrogen receptor agonist G-1 has also shown promise by disrupting phosphoproteomic features associated with sunitinib resistance, including the PI3K-AKT and other pathways. This suggests that targeting GPER could offer novel strategies to overcome sunitinib resistance in RCC 76. However, a comparative study of tamoxifen monotherapy versus tamoxifen combined with IL-2/IFN-α in advanced RCC patients showed no significant therapeutic advantage for the combination. Despite this, the potential for ER-targeted therapies in RCC, particularly in overcoming TKI resistance, remains promising 120. Estrogen receptor-targeted therapies hold great potential for combination treatment in RCC, especially in addressing sunitinib resistance. Although certain combination regimens have not demonstrated significant clinical benefits, these studies highlight new directions for RCC treatment strategies and provide robust theoretical support for future clinical applications.

#### 3.3.2 Current Status of Clinical Trials of Estrogen Receptor Antagonists in Kidney Cancer

With increasing research on estrogen receptors in RCC, studies on estrogen receptor antagonists for RCC treatment are gradually progressing. SERMs, such as tamoxifen and raloxifene, exert their effects by blocking estrogen action. On the other hand, new ER-targeted drugs, such as SERDs like fulvestrant, work by blocking and degrading the estrogen receptor.

When used in combination therapy, the effects are more promising. In a study involving 10 advanced RCC patients, combination therapy of tegafur and tamoxifen resulted in 1 complete response (CR) and 3 partial responses (PR), achieving an overall response rate of 40%. Notably, 1 out of 2 ER-positive tumors showed a therapeutic effect, and 3 out of 4 ER-negative tumors responded well, suggesting that while the ER status can be a reference, it is not the sole predictor of therapeutic response 118. From a pharmacological perspective, tamoxifen may not be the optimal ERβ antagonist. In an *in vivo* study, more selective ERβ antagonists like ICI 182,780 and PHTPP reduced the tumor weight of ERβ-positive RCC by 40.3% and 51%, respectively, significantly higher than tamoxifen's 17.8% 72. This suggests that optimizing the choice of drugs targeting ERβ could be a key pathway to improving efficacy. Furthermore, new SERDs, such as fulvestrant, have demonstrated strong receptor degradation and anti-proliferative effects in breast cancer, although their application in RCC is not yet widespread, offering an important direction for future drug development. Additionally, raloxifene has shown potential in inhibiting the proliferation and migration of RCC cells with high expression of histone demethylase LSD1, suggesting that ER-related pathways may work synergistically with epigenetic targets, thereby expanding the therapeutic possibilities 121.

In summary, although current clinical studies have not demonstrated widespread efficacy of tamoxifen-like ER modulators in RCC, preliminary signals in specific subgroups and combination therapies, along with the mechanistic advantages and prospects of new ERβ antagonists and SERDs, suggest that further research on these drugs in RCC holds scientific and clinical value.

Table 1 lists various targeted estrogen receptor drugs at different stages of clinical trials, including Drug Name, Trial Name, Phase in the Clinical Trial, Sample Size, Cancer Type, MoA / Target, and Primary Outcome.

## 4. Challenges and Future Prospects of Estrogen-Targeted Therapy

### 4.1 Selective Regulation of Estrogen Receptors

#### 4.1.1 Functional Differences Between ERα and ERβ in Different Tumor Types

When examining the roles of ERα and ERβ in cancer, it becomes evident that these two receptors play distinct roles across different tumor types. In breast cancer, ERα is closely associated with cell proliferation and tumor progression. It promotes cancer cell growth by inducing the expression of MYC and cyclin D1, thereby driving cell cycle progression 122. Additionally, ERα plays a critical role in inflammation and tumor development. For example, ERα activation in neonatal mouse prostates promotes proliferation and infiltration of inflammatory cells, which are strongly linked to tumor development 123. In contrast, ERβ expression in breast cancer cells suppresses the expression of ERα target genes and is associated with improved prognosis and enhanced responsiveness to endocrine therapy.

In the context of PCa, ERα expression is associated with the proliferation and multilayering of prostate epithelial cells 124, while ERβ is linked to the suppression of epithelial-to-mesenchymal transition (EMT) and reduced invasiveness. Studies have shown that ERβ expression is decreased in high-invasive prostate cancer cells, and activation of ERβ can inhibit EMT in prostate cancer, indicating that ERβ plays a critical role in inhibiting tumor progression. In BCa, ERβ becomes the predominant estrogen receptor subtype, and its role varies across different stages of the tumor. Although most studies indicate that high ERβ expression correlates with high-grade or muscle-invasive tumors, suggesting its potential oncogenic role in certain conditions, ERβ can also suppress tumor invasion by regulating cell adhesion molecules and migration-related pathways under the influence of specific drugs or agonists. In contrast, ERα expression in bladder cancer is more restricted, predominantly observed in low-grade, non-muscle invasive tumors. Its role in regulating cell cycle, chemokines, and AKT signaling displays strong context-dependent effects.In RCC, ERβ is considered the most active subtype in most studies, exhibiting functional patterns distinct from those observed in PCa and BCa. In RCC, activation of ERβ is often associated with the upregulation of cancer stem cell phenotypes, angiogenesis, and metastatic potential. In contrast, although ERα expression is relatively low in RCC, in specific subtypes such as Xp11.2 translocation tumors, it may contribute to the formation of genomic instability by regulating DNA repair mechanisms. ERα expression can also increase due to VHL (Von Hippel-Lindau) gene deletion, indirectly activating oncogenic signals.

A further comparison of PCa, BCa, and RCC reveals notable differences in the role of estrogen receptors in these urological cancers. In PCa, ERβ typically acts as a tumor suppressor by inhibiting cell proliferation, EMT, and metastasis, and its agonists effectively slow tumor progression. In contrast, ERβ in BCa exhibits a more complex function: at different stages of cancer, ERβ can promote cell proliferation and invasion while also exhibiting antitumor effects through upregulation of E-cadherin and suppression of EMT, highlighting its regulatory role in the tumor microenvironment. Unlike PCa and BCa, ERβ in RCC is more often associated with tumor-promoting mechanisms, particularly in angiogenesis, cancer stem cell phenotype maintenance, and metastasis. ERβ activation can enhance tumor invasion and drug resistance by modulating specific non-coding RNAs and cytokine networks. However, ERα tends to promote tumor progression across these three cancers, particularly in PCa and RCC, where ERα facilitates tumor growth through enhanced cell proliferation, migration, and angiogenesis pathways.

Research on ER in other tumor types has further revealed the tissue-specific dual functions of estrogen receptor signaling. In colorectal cancer, ERβ suppresses tumor progression by downregulating MYC and cyclin E, as well as inducing cell cycle inhibitors such as p21 and p27, whereas ERα expression is associated with poor prognosis 125. Similarly, in gynecologic malignancies, distinct patterns of ER expression reflect markedly different biological behaviors—high ERα expression in ovarian cancer often predicts favorable outcomes, while ERβ upregulation correlates with enhanced lymph node metastasis and disease progression 126. These observations reinforce the notion that ERα and ERβ subtypes can exert context-dependent, and sometimes opposing, roles in tumor biology, highlighting the necessity of elucidating subtype-specific functions and signaling crosstalk to optimize estrogen receptor-targeted therapeutic strategies.

In summary, the functional differences between ERα and ERβ across cancer types are profound, as summarized in Table 2. These distinctions not only provide critical insights for personalized cancer therapies but also emphasize the importance of developing subtype-selective estrogen receptor modulators. ERβ activation is generally associated with tumor suppression, while ERα can, in certain contexts, promote tumor growth. These findings hold significant potential for advancing safer and more effective treatment strategies, particularly for breast, prostate, and colorectal cancers.

### 4.2 Resistance and Limitations of Estrogen-Targeted Therapy

#### 4.2.1 Mechanisms of Resistance to Estrogen Receptor-Targeted Therapy

**Figure 4:**
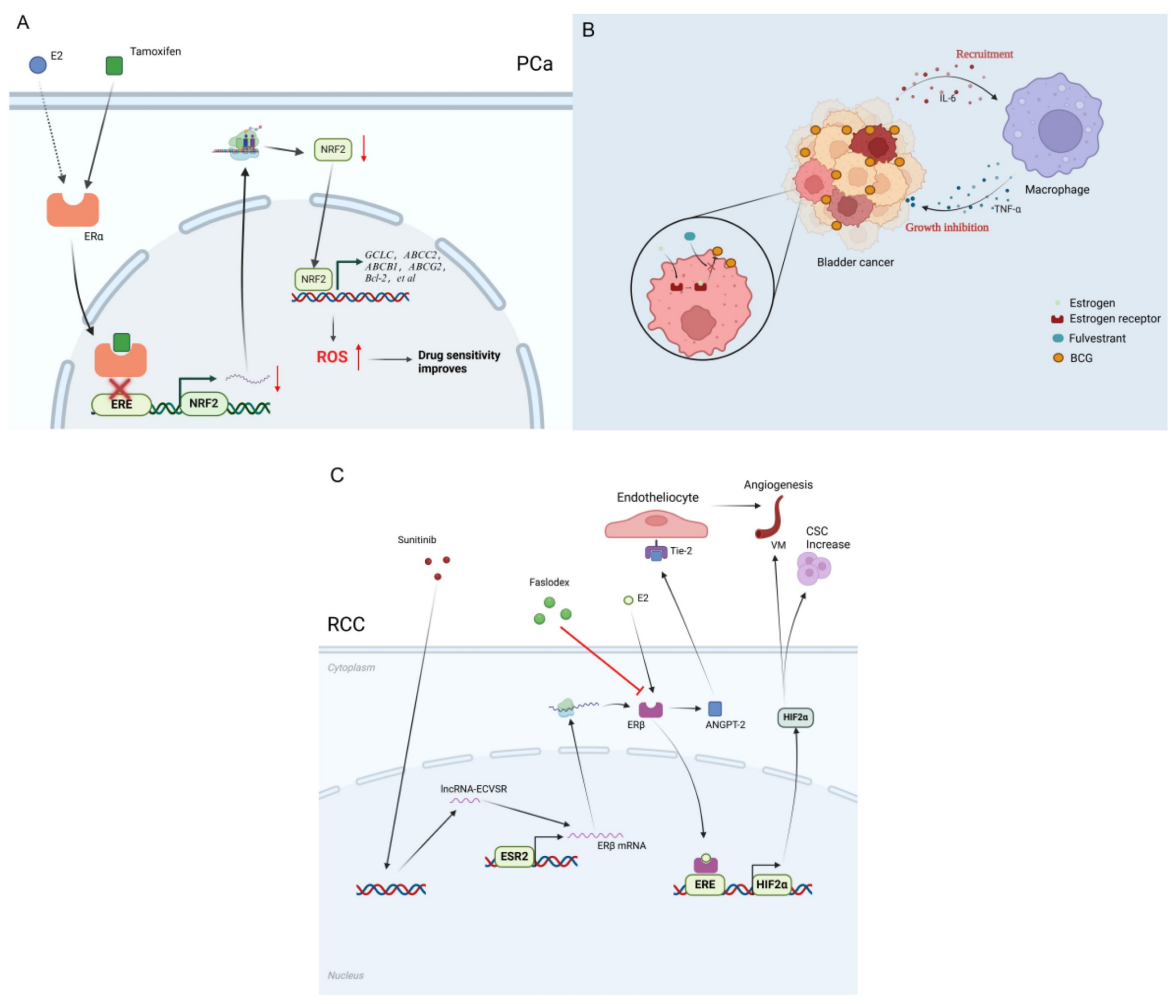
** Estrogen receptor-mediated mechanisms influencing therapy responses in urological tumors.** a. Tamoxifen competitively binds to estrogen receptor alpha, inhibiting the binding of estrogen receptors to ERE, thereby reducing the transcription and translation of the NRF2 gene. The decrease in NRF2 protein leads to the downregulation of antioxidant genes (such as GCLC) and multidrug resistance-related genes (such as ABCC2, ABCB1, Bcl-2, etc.). The accumulation of ROS results in enhanced oxidative stress in cells, making cancer cells more sensitive to enzalutamide due to oxidative damage. b. Estrogen can reduce the adhesion and internalization of BCG in bladder cancer cells through estrogen receptors, while also inhibiting the recruitment of monocytes and macrophages, thus diminishing the efficacy of BCG. Fulvestrant can counteract this effect of estrogen and promote the release of IL-6 from bladder cancer tissue to recruit monocytes/macrophages, which in turn can secrete TNF-α to inhibit the growth of bladder cancer cells. c. Sunitinib can upregulate the expression of lncRNA-ECVSR, enhancing the stability of ERβ mRNA, thereby promoting the expression levels of ERβ. ERβ binds to the estrogen response elements (ERE) in the promoter region of HIF2α, upregulating HIF2α expression, which ultimately promotes the characteristics of cancer stem cells (CSCs) and the formation of VM (vasculogenic mimicry). Additionally, ERβ can regulate angiogenesis through the ANGPT-2/Tie-2 signaling pathway. Faslodex can target ERβ, inhibiting the ERβ/ANGPT-2/Tie-2 pathway, and enhance the therapeutic effect of sunitinib on RCC (Renal Cell Carcinoma).

Targeted therapies against the ER in genitourinary tumors face substantial barriers to clinical translation, primarily due to complex mechanisms of therapeutic resistance that limit long-term efficacy. As illustrated in Figure 4, ER signaling modulates the response of prostate, bladder, and renal cancers to endocrine, immune, and targeted therapies. Unlike in breast cancer, the expression patterns and functional roles of ERs in genitourinary tumors malignancies (such as prostate, bladder, and renal carcinomas) differ markedly 127. Tumor heterogeneity, together with the intricate crosstalk between ER signaling and pathways such as PI3K/Akt/mTOR and MAPK, generates multilayered compensatory networks that undermine the sustained effects of single-agent ER-targeted therapy 128. Consequently, overcoming resistance has become a pivotal focus in therapeutic development.

Although ER serves as a major therapeutic target, both innate and acquired resistance frequently emerge during ER-targeted therapy, largely driven by alterations in ER expression and function. Acquired mutations within the ligand-binding domain (LBD) of the ESR1 gene—such as D538G, Y537S, E380Q, and L536R—constitute primary drivers of hormone-independent tumor growth 129. These mutations induce conformational rearrangements in ER that sustain receptor activity even in the absence of estrogen, thereby maintaining downstream transcriptional programs. Proteomic analyses have revealed that these mutations hyperactivate key signaling cascades including CDK, mTOR, and MAPK, and remodel phosphorylation networks that reinforce proliferative and survival advantages 130. Moreover, mutant ER can modulate microRNA expression, such as the upregulation of miR-301b, which enhances PRKD3 expression and further amplifies oncogenic signaling 131.

Such conformational changes also reduce the binding affinity of ER antagonists, necessitating higher drug concentrations for effective inhibition. However, agents like fulvestrant suffer from poor oral bioavailability, making it challenging to achieve therapeutically relevant receptor saturation *in vivo*.

In prostate cancer, ERβ splice variants (β2 and β5) stabilize HIF-1α and HIF-2α, thereby activating hypoxia-responsive signaling and inducing genes such as Notch3, ABCG2, and MDR1, which promote stem-like properties and chemoresistance 28. This mechanism parallels the survival signaling induced by mutant ERα. Concurrently, cross-activation between ER signaling and the PI3K/AKT and MAPK pathways allows cancer cells to maintain proliferation and resistance under conditions of androgen deprivation or ER downregulation 132. Overexpression of coactivator SRC-3 or loss of transcriptional corepressors NCOR1/2 further disrupts ER-mediated transcriptional balance, diminishing the efficacy of antiestrogen therapies 133.

Additionally, bicalutamide treatment can upregulate ERα, activating the NRF2 signaling axis, which enhances anti-apoptotic capacity and oxidative stress responses while inducing the stem cell marker CD44, thereby establishing the ERα-NRF2-CD44 axis that markedly reduces drug-induced cell death 88. Meanwhile, aromatase-mediated endogenous estrogen promotes ERα binding to the MMP12 promoter, increasing MMP12 expression. MMP12 cooperates with CD44 to drive tumor metastasis and progression to CRPC 134, collectively augmenting cellular migration, invasion, and therapeutic resistance.

The non-classical ER signaling pathways also play a pivotal role in CRPC. Following androgen deprivation, GPER1 expression is upregulated, and its agonist G-1 has been shown to induce neutrophil-associated necrosis and inhibit tumor growth 135. In contrast, the ER degrader fulvestrant restores cytolytic responsiveness in resistant models characterized by phenotypic plasticity and reduced NK cell sensitivity 89. Collectively, these findings indicate that ER signaling not only regulates estrogen-dependent proliferation but also modulates immune evasion and cell fate decisions within therapy-resistant tumor contexts.

In RCC, ERβ regulates autophagy and M2 macrophage polarization, activating the ARG1/ERβ/p-AKT axis and thereby reducing sensitivity to tyrosine kinase inhibitor (TKI) therapy 136. Moreover, ERβ induces ANGPT-2 expression and promotes Tie-2 phosphorylation, enhancing angiogenesis and contributing to sunitinib resistance 74. Conversely, activation of GPER1 by G-1 suppresses ATF2, mTOR, and PLK signaling pathways, impairing DNA repair and mitotic progression, effectively reversing TKI resistance 76. From an immunological perspective, the ERβ agonist LY500307 decreases MDSC infiltration and improves responsiveness to anti-PD-1 therapy 110.

In bladder cancer, cancer-associated fibroblasts (CAFs) within the tumor microenvironment activate ERβ signaling through IGF-1, leading to Bcl-2 upregulation and the induction of cisplatin resistance 137. Tamoxifen enhances chemosensitivity to methotrexate and vinblastine through a non-classical, MDR1-independent ER pathway. Mechanistically, tamoxifen resistance involves SRC-3 overexpression, HER2 amplification, and aberrant activation of transcription factors such as FOXA1 and c-Myc, collectively driving uncontrolled proliferation and reduced therapeutic responsiveness 138.

#### 4.2.2 Strategies to Overcome Resistance to Estrogen Receptor-Targeted Therapy

To overcome these resistance mechanisms, researchers have developed next-generation SERDs and proteolysis-targeting chimeras (PROTACs), which more effectively degrade both wild-type and mutant ERα, improve pharmacokinetic properties, and enhance therapeutic efficacy 139. In parallel, strategies targeting ER-DNA binding or disrupting its interactions with co-factors have shown potential to inhibit ligand-independent ER activity 129.

Given that resistance in the ER pathway is often accompanied by aberrant activation of bypass signaling, combination strategies involving pathway inhibitors have become a focus of investigation. For instance, the combined use of PI3K/AKT/mTOR and CDK4/6 inhibitors can simultaneously block ER activation and cell cycle progression, promoting apoptosis and restoring drug sensitivity, as validated in multiple clinical trials 132. In RCC, inhibition of downstream effectors of ERβ/GPER1, such as mTOR, ATF2, and PLK, can significantly reverse TKI resistance 76, while HDAC inhibitors can reactivate hormone therapy responsiveness by restoring methylation-silenced ER expression 140. In bladder cancer (BCa) exhibiting ERβ-dependent resistance, blockade of the IGF-1/ERβ/Bcl-2 axis markedly reverses resistance in both *in vitro* and *in vivo* models, highlighting the potential therapeutic value of this pathway 137.

Moreover, strategies targeting the TME have garnered increasing attention. Factors secreted by CAFs can promote tumor stemness and EMT phenotypes through activation of the Notch and Wnt pathways, creating a “protective niche.” Accordingly, targeting microenvironmental factors such as IGF-1, CSF1/CSF1R, and ANGPT-2 can enhance responses to immunotherapy and TKIs. For example, in RCC, blockade of CSF1/CSF1R signaling or ANGPT-2 enhances responses to PD-1 antibodies and TKI therapy 74,110.

Overall, overcoming ER-related resistance in urological tumors requires a multi-layered approach that integrates next-generation ER degraders, co-inhibition of signaling pathways, and modulation of the tumor microenvironment, with the goal of achieving more durable therapeutic responses and improved clinical outcomes.

### 4.3 Future Estrogen-Targeted Therapy Strategies

#### 4.3.1 Development of New Estrogen Receptor Modulators

In recent years, advancements in understanding ER signaling pathways have driven breakthroughs in the development of next-generation estrogen receptor modulators. These novel agents overcome the limitations of traditional SERMs and SERDs, exhibiting greater selectivity, improved tolerability, and potential efficacy in endocrine-resistant cancers. These innovative therapies are reshaping the treatment landscape of genitourinary tumors.

Among the new oral SERDs, elacestrant and giredestrant have emerged as pioneers. The FDA recently approved elacestrant for the treatment of breast cancer. Compared with traditional SERDs such as fulvestrant, elacestrant exhibits superior absorption, a longer duration of action, favorable pharmacokinetic properties, and stronger ER inhibition 141. At higher doses, elacestrant binds to the estrogen receptor, induces conformational changes, and promotes receptor degradation, thereby exhibiting potent antitumor activity. Meanwhile, at lower doses, it exerts estrogen-like effects, which can help alleviate hot flashes and prevent bone resorption, mitigating certain adverse effects associated with endocrine therapy. Another novel SERD, giredestrant, also demonstrates excellent antitumor potency. It interacts with the ERα protein through multiple binding modes and has been structurally optimized to improve physicochemical properties, enabling effective oral administration at low doses 142.

Elacestrant is the first oral SERD to achieve success in a phase III clinical trial, particularly showing significant efficacy and good tolerability in tumors harboring ESR1 mutations. In the EMERALD trial (NCT03778931), elacestrant monotherapy demonstrated superior efficacy and safety compared with standard endocrine therapy—including fulvestrant or aromatase inhibitors—in postmenopausal women and men with ER+/HER2- advanced or metastatic breast cancer, irrespective of ESR1 mutation status 143. Giredestrant has also been investigated in multiple preclinical and clinical studies. The acelERA trial, a randomized, phase II, open-label, multicenter study, compared giredestrant monotherapy with standard endocrine therapy in patients with ER+/HER2- locally advanced or metastatic breast cancer. Although the primary endpoint, investigator-assessed progression-free survival (INV-PFS), did not reach statistical significance, giredestrant showed consistent therapeutic benefits across key subgroups, with higher response and clinical benefit rates observed among patients with ESR1-mutant tumors 144. Furthermore, ongoing clinical studies are evaluating the efficacy of giredestrant combined with palbociclib versus letrozole plus palbociclib in ER+/HER2- locally advanced or metastatic breast cancer, as well as giredestrant versus adjuvant endocrine therapy in early-stage ER+/HER2- breast cancer.

Researchers have also developed SERDs with acrylic side chains 145, such as G1T48 and AZD9496, which employ unique chemical modifications to avoid cross-resistance with other hormone receptors. These compounds retain activity across a broader spectrum of endocrine-resistant tumor models and show great promise when combined with CDK4/6 inhibitors. To further enhance SERD efficacy, some novel agents incorporate basic amino side chains, such as camizestrant and imlunestrant. These structural optimizations improve oral absorption and ERα degradation capabilities, paving the way for new combination therapy strategies.

Beyond ERα, the development of selective ERβ agonists has garnered considerable attention. Compounds such as diarylpropionitrile and liquiritigenin have been identified as ERβ-selective activators, offering unique therapeutic advantages for hormone-dependent cancers 146. ERβ exhibits distinct biological functions compared to ERα across various tissues, and its activation can effectively reduce the side effects commonly associated with ERα-targeted therapies. ERβ-selective agonists may hold particular promise in genitourinary tumors, as ERβ is highly expressed in prostate cancer tissues and represents a potential therapeutic target better suited for this malignancy.

Ormeloxifene, a next-generation SERM primarily used for contraception, has shown promising anticancer activity in various hormone-dependent tumors. *In vitro* and *in vivo* studies suggest that ormeloxifene induces apoptosis and inhibits cell proliferation, demonstrating strong anticancer potential in estrogen-dependent prostate cancer. While clinical data remain limited, preliminary results suggest new applications for ormeloxifene in male genitourinary cancers 96.

Combining the advantages of SERMs and SERDs, the development of SERM/SERD hybrids has become a hot research topic. Lasofoxifene, a leading example of this class, combines ER antagonism with degradation mechanisms, offering enhanced antitumor activity and tissue selectivity 145. Studies indicate that these agents not only provide higher therapeutic efficacy but also reduce hormone-related adverse effects while maintaining bone density. Researchers are further optimizing next-generation estrogen receptor modulators by exploring the potential of non-canonical ER signaling pathways. These novel drugs not only function via the classical EREs pathway but also interact with transcription factors such as NF-κB and Sp1 to regulate cancer-related gene expression. This multi-target drug design strategy holds promise for developing anticancer therapies with greater specificity and fewer side effects.

#### 4.3.2 Potential of Combining Estrogen-Targeted Therapy with Other Treatments

While ER-targeted therapies and combination regimens have traditionally been extensively studied and applied in breast cancer treatment, recent research has begun to explore their potential roles in genitourinary tumors. These studies suggest that combining ER-targeted therapies with other agents could provide new strategies to overcome resistance in genitourinary malignancies.

Studies indicate that combining SERDs/SERMs with AR inhibitors, such as enzalutamide, may effectively suppress the proliferation and survival of prostate cancer cells 147. Additionally, palbociclib, a CDK4/6 inhibitor, when used in conjunction with ER-targeted therapies, has been shown to delay the progression of CRPC. This combination not only effectively inhibits cell cycle progression but may also overcome resistance to single-agent AR inhibitors by downregulating ER signaling pathways.

The crosstalk between ER signaling and the PI3K/Akt/mTOR pathway has been well-documented in various genitourinary tumors. Combination therapies targeting these pathways have demonstrated strong antitumor activity. For example, the mTOR inhibitor everolimus combined with ER degraders has shown potential to inhibit tumor growth in ER-expressing renal and bladder cancer models 148.

Preclinical studies have revealed that combining PI3K inhibitors, such as alpelisib, with SERDs/SERMs can significantly reduce the proliferation of genitourinary tumor cells, particularly in cases resistant to conventional therapies. These combination strategies hold promise for overcoming resistance and extending progression-free survival by inhibiting multiple signaling pathways simultaneously 149.

In advanced bladder and kidney cancers, CDK4/6 inhibitors like palbociclib have demonstrated potential when combined with ER-targeted therapies. This combination effectively blocks cancer cell cycle progression and reduces proliferation. Research shows that combining SERDs with palbociclib significantly suppresses tumor growth and prolongs progression-free survival in patients with resistant cancers 150.

Recent studies have also investigated combining ER-targeted therapies with inhibitors of the Notch, ERK, and PI3K signaling pathways. These combinations have shown promise in overcoming tumor resistance in breast cancer and may exhibit similar benefits in bladder and kidney cancers. Although research in genitourinary tumors is still in its early stages, preliminary findings suggest that such combination therapies could play a significant role in these malignancies.

Despite being in its infancy, the exploration of ER-targeted therapies in genitourinary tumors highlights the growing potential of combining these therapies with other pathway inhibitors, such as PI3K, CDK4/6, and mTOR inhibitors. Future clinical trials are expected to further validate the efficacy of these combinations in treating genitourinary tumors, providing new therapeutic options for patients, especially those resistant to standard therapies. These combination therapies could not only delay tumor progression but also improve overall survival and quality of life for patients.

#### 4.3.3 Estrogen Modulation Strategies in Personalized Medicine

Precision medicine strategies for PCa and other malignancies are increasingly shifting toward personalized and targeted therapies. For breast cancer, treatment regimens are typically determined based on the expression patterns of ERα, progesterone receptor (PR), and HER2^151^. Similarly, therapies for non-small cell lung cancer (NSCLC) are guided by mutations in genes such as EGFR, KRAS, or ALK^152^. However, precision medicine based on gene expression profiling has yet to become widely adopted in prostate cancer. Recently, growing research efforts have focused on employing molecular diagnostic tools such as immunohistochemistry (IHC), fluorescence *in situ* hybridization (FISH), and DNA, RNA, and micro-RNA analyses to enhance treatment accuracy for PCa patients.

The heterogeneity of ER determines their varying roles and therapeutic responses across tumor types. Precision medicine emphasizes tailoring treatment strategies based on individual molecular characteristics. For example, analyzing the expression patterns of ERα and ERβ through IHC or liquid biopsy can enable the customization of treatment plans for patients with prostate, bladder, and kidney cancers. Targeted therapies based on ERβ, in combination with existing AR inhibitors or chemotherapeutic agents, may further improve therapeutic outcomes and mitigate the development of resistance.

Emerging studies indicate that selective ERβ agonists not only inhibit tumor cell growth but also modulate the immune microenvironment by regulating pathways such as NF-κB and interleukin signaling. These effects provide anti-inflammatory and immunomodulatory benefits, suggesting that integrating ER-targeted therapy with immunotherapy could further enhance overall treatment efficacy and prolong disease-free survival for patients.

Despite the promising potential of subtype-selective ER-targeted therapies in genitourinary tumors, several challenges remain in clinical application. First, the high heterogeneity of ER expression patterns in patient tumors necessitates precise molecular diagnostics prior to treatment. Second, the currently available ER subtype-selective drugs for genitourinary tumors are limited, highlighting the need for more preclinical studies and clinical trials to evaluate the long-term safety and efficacy of these therapies.

## 5. Future Outlook

Recent advances in ER-targeted therapy have provided novel insights into the management of urological tumors, highlighting both mechanistic depth and translational potential. Emerging evidence underscores the multifaceted role of ER signaling in tumor biology, ranging from its regulation of lipid metabolism and ferroptosis to its influence on immune responses and therapeutic resistance. For instance, 27-hydroxycholesterol (27-HC), a selective SERM, can modulate ER and Liver X Receptor (LXR) signaling, promoting proliferation in prostate cancer while offering potential intervention points for genitourinary tumors 153,154. Meanwhile, ER-mediated suppression of ferroptosis via MBOAT1 upregulation presents a promising therapeutic target—especially when ER antagonists such as fulvestrant are combined with ferroptosis inducers to overcome hormone resistance 155. Furthermore, phytoestrogens and hybrid compounds derived from natural products, including isoflavones and artemisinin derivatives, demonstrate inhibitory activity against prostate cancer, emphasizing the potential of structure-based drug design for selective ER modulation 156,157.

From a clinical perspective, ER-targeted strategies that have transformed ER-positive breast cancer treatment—particularly through SERMs and SERDs—are now being adapted for urological cancers. Oral SERDs such as elacestrant and giredestrant have shown improved bioavailability and tolerability, and early studies suggest that combining ER blockade with androgen deprivation therapy could yield synergistic effects in prostate cancer 158,159. These developments offer a translational framework for optimizing endocrine-based therapies in genitourinary tumors.

Looking ahead, several next-generation modalities are reshaping the landscape of ER-targeted treatment. Nanoparticle-based delivery systems have shown remarkable potential to enhance drug bioavailability and tumor selectivity. For example, PSMA-targeted PLGA-PEG nanoparticles loaded with the ERα blocker toremifene significantly increased intratumoral concentration and induced extensive necrosis, validating nanoparticle-enhanced ER inhibition as a feasible clinical direction 160. Concurrently, targeted protein degradation technologies, particularly PROTACs such as ARV-471, demonstrate efficient ERα degradation with favorable safety and durable clinical activity in endocrine-resistant breast cancers, suggesting a transferable therapeutic strategy for ER-positive urological malignancies 161.

Emerging degradation-based and RNA-targeted platforms, including LYTAC, RIBOTAC, and siRNA/ASO approaches, further broaden the therapeutic horizon by enabling precise modulation of ER expression at the protein and transcript levels 141,162. These novel technologies could complement conventional SERMs and SERDs, forming a multi-layered intervention system capable of overcoming resistance and improving treatment precision.

However, several critical challenges must be addressed before these innovations can achieve clinical translation. Spatial heterogeneity of ER expression within prostate and renal tumors necessitates refined molecular subtyping and diagnostic co-development. Delivery systems must balance efficiency with safety, minimizing off-target accumulation in organs such as the liver and kidney. Additionally, the pharmacokinetic complexity and manufacturing scalability of PROTACs remain limiting factors for large-scale clinical adoption. Future research should therefore focus on optimizing ER-targeted strategies through integrated approaches—combining receptor degradation, precision delivery, and biomarker-guided patient selection—to realize personalized endocrine therapy for urological cancers.

In summary, the future of ER-targeted therapy lies in a paradigm shift toward precision, adaptability, and cross-platform integration. By uniting molecular degradation, nanotechnology, and diagnostic stratification, ER-based interventions may evolve from experimental strategies into clinically robust, patient-specific treatments for genitourinary malignancies.

## 6. Conclusion

The estrogen signaling network has emerged as a pivotal but underappreciated regulator in the pathogenesis, heterogeneity, and therapeutic responsiveness of urological malignancies. Beyond its traditional role in reproductive biology, estrogen receptor (ER) activity—mediated by ERα, ERβ, and GPER—shapes the molecular and cellular landscape of prostate, bladder, and renal cancers through intricate genomic and non-genomic mechanisms. These receptors function not as isolated hormonal sensors but as dynamic molecular integrators that coordinate proliferative, apoptotic, metabolic, and immune processes within the tumor microenvironment.

ERα primarily exhibits oncogenic potential by activating MAPK, PI3K/AKT, and β-catenin signaling, thereby promoting proliferation, epithelial-mesenchymal transition (EMT), and therapeutic resistance. In contrast, ERβ, particularly its classical isoform ERβ1, exerts tumor-suppressive functions through transcriptional regulation of PTEN, inhibition of HIF-1α, induction of cell-cycle arrest, and attenuation of inflammatory signaling. However, variant isoforms such as ERβ2 and ERβ5 can acquire tumor-promoting properties under hypoxic or stem-like conditions, fostering invasiveness, metabolic reprogramming, and chemoresistance. Meanwhile, GPER bridges nuclear and membrane signaling domains, mediating rapid non-genomic effects. Depending on the cellular and metabolic context, GPER may either suppress tumor cell growth or enhance migration and angiogenesis through the PI3K/AKT/MMP axis.

Across urological cancers, ER signaling displays tumor-type-specific behavior. In prostate cancer, ERβ functions as a counter-regulator of androgen receptor (AR) signaling, providing a potential avenue to circumvent castration resistance, whereas ERα and its splice variant ERα-36 drive metastasis and endocrine resistance. In bladder cancer, ERα and ERβ exhibit an inverted dichotomy—ERα is associated with protective effects in early-stage disease via INPP4B-mediated AKT suppression, whereas ERβ promotes progression, recurrence, and immune evasion through inflammatory and noncoding RNA-dependent pathways. In renal cell carcinoma, ERβ orchestrates angiogenesis, cancer stemness, and vasculogenic mimicry through lncRNA and circRNA regulatory networks, while ERα-36 correlates strongly with metastasis and poor prognosis. These findings collectively depict ER signaling as a context-dependent molecular switch that dynamically integrates hormonal, epigenetic, and microenvironmental cues to govern tumor evolution.

Therapeutically, selective estrogen receptor modulators (SERMs), selective estrogen receptor degraders (SERDs), and GPER-targeted compounds are gaining recognition as promising anti-tumor strategies in urological oncology. Agents such as tamoxifen, raloxifene, and fulvestrant have demonstrated the ability not only to inhibit ER-driven proliferation but also to remodel the immune landscape by enhancing NK- and T-cell infiltration while reducing immunosuppressive cell populations. Combination regimens that integrate ER-targeted therapy with androgen blockade, tyrosine kinase inhibitors (TKIs), or immune checkpoint inhibitors may further overcome therapeutic resistance and improve outcomes. Mechanistically, the intersection of ER signaling with the AR, HIF, and PI3K/AKT pathways offers a strong biological rationale for multi-targeted therapeutic designs.

Future research should focus on precision stratification and network-level modulation of ER signaling. Comprehensive profiling of receptor subtypes, splice variants, and signaling dependencies will be crucial to guide personalized treatment decisions. Moreover, unraveling the crosstalk between ER signaling and immune metabolism, angiogenesis, and noncoding RNA regulation may uncover novel vulnerabilities for therapeutic intervention. Incorporating these insights into biomarker development and clinical trial design will redefine the hormonal paradigm of urological oncology. Ultimately, estrogen receptors should no longer be regarded as peripheral participants but as central, targetable nodes within an interconnected oncogenic network, holding transformative potential for precision medicine in prostate, bladder, and renal cancers.

## Figures and Tables

**Figure 3:**
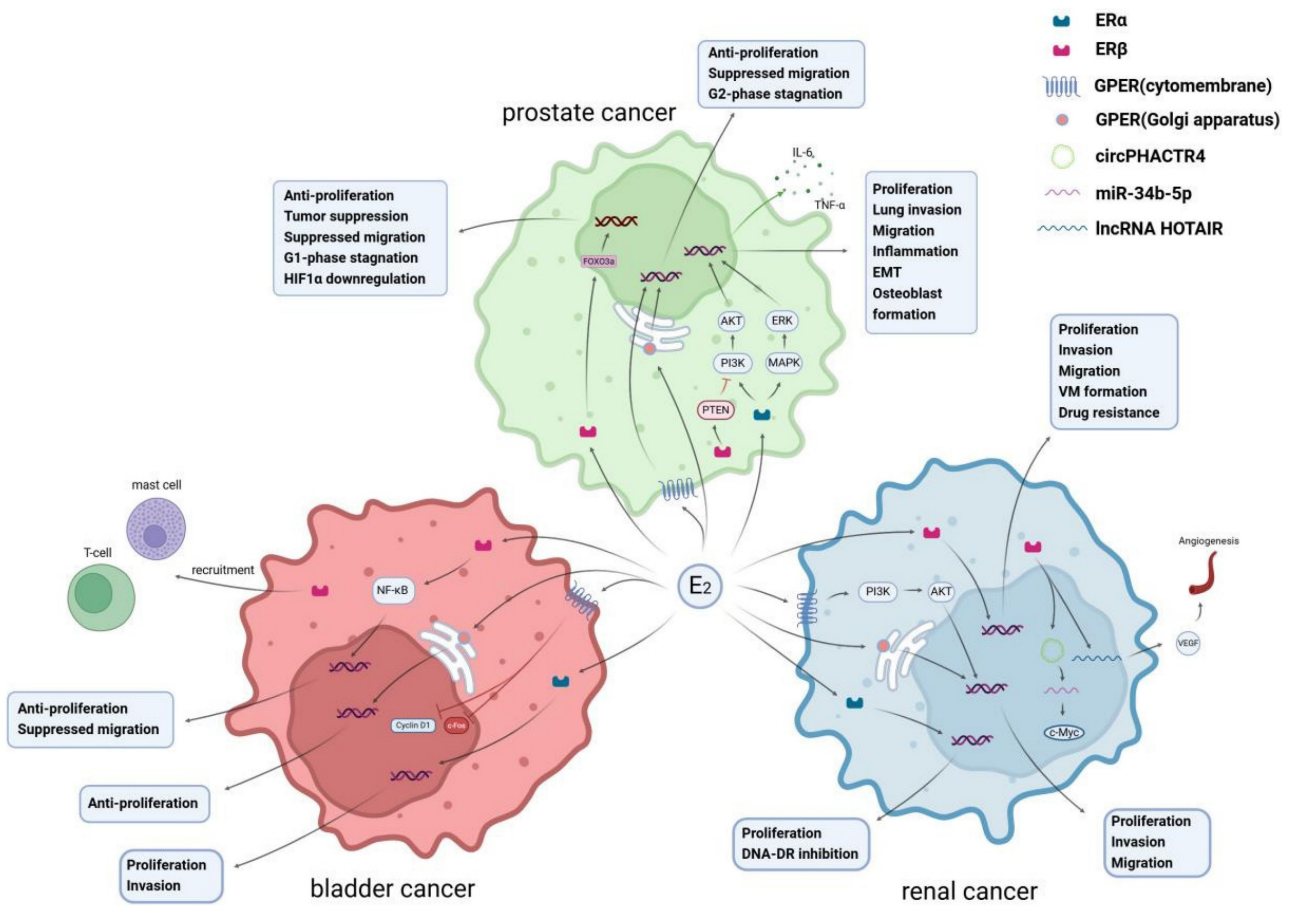
** Biological roles of estrogen receptors.** Estrogen exerts different biological effects in prostate cancer, bladder cancer, and renal cancer through ERα, ERβ, and GPER, respectively. In prostate cancer, E2 activates the PI3K/AKT and MAPK/ERK pathways through ERα, which can promote cell proliferation and migration. It can also enhance inflammation and microenvironmental remodeling by upregulating the expression of IL-6 and TNF-α. ERβ, on the other hand, can inhibit the PI3K/AKT pathway through PTEN, reducing drug resistance, and activate FOXO3a to upregulate apoptosis-regulating genes, inducing cell apoptosis. In bladder cancer, ERβ activates the NF-κB signal to promote cell proliferation and can also recruit mast cells and T cells to affect tumor progression. GPER rapidly activates non-genomic signaling pathways, inhibiting Cyclin D1 and c-Fos, reducing proliferation and invasion. In renal cell carcinoma, E2 activates lncRNA HOTAIR and VEGF through ERβ, upregulating angiogenesis-related pathways, and can also regulate tumor stem cell phenotype (CSC) through related RNA, enhancing migration and self-renewal capabilities.

**Table 1:** Completed and Ongoing Clinical Trials of Targeted Estrogen Receptor Drugs in Urological Cancers

Drug name	Trial name/NCT No	Phase	N	Cancer Type	MoA / Target	Primary outcome	References
Tamoxifen	Study of tamoxifen in metastatic renal cell carcinoma and the influence of certain prognostic factors	II	79	RCC	ER	ORR	82
Genistein	Efficacy and Safety of Short-Term Genistein Intervention in Patients with Localized Prostate Cancer Prior to Radical Prostatectomy	II	54	PCa	ER	serum PSA levels	91
Phytoestrogen	Effects of a Phytoestrogen Intervention and Estrogen Receptor β Genotype on Prostate Cancer Proliferation and PSA Concentrations	II	140	PCa	ER	tumor proliferation	92
Toremifene	Phase IIA clinical trial to test the efficacy and safety of Toremifene in men with high-grade prostatic intraepithelial neoplasia	IIA	21	high-grade PIN	ER	safety and efficacy	93
Toremifene	Toremifene for the Prevention of Prostate Cancer in Men With High Grade Prostatic Intraepithelial Neoplasia	IIB	512	high-grade PIN	ER	incidence of prostate cancer	94
Fulvestrant	Phase II study of fulvestrant (Faslodex) in castration resistant prostate cancer	II	20	CRPC	ER	TTP	97
Fulvestrant	Experience with fulvestrant acetate in castration-resistant prostate cancer patients	II	7	CRPC	ER	PSA response	98
Tamoxifen	Evaluation the Treatment of Tamoxifen of Low/​Intermediate Risk Bladder Tumors (NCT02197897)	II	15	BCa	ER	Clinical response	110
Genistein	Study of Genistein in Reducing Side Effects of Superficial Bladder Cancer Treatment (NCT01489813)	II	36	BCa	ER	Change in severity of urinary symptoms	111
Tamoxifen	Combined chemoendocrine treatment with tegafur and tamoxifen for advanced renal cell carcinoma	II	10	advanced RCC	ER	ORR	117

TTP, Time to Progression; PSA, Prostate-Specific Antigen; ORR, Objective Response Rate

**Table 2:** The Role of Estrogen Receptors and GPER in Tumor Progression, Therapeutic Impacts, and Clinical Prospects

Tumor Type	Estrogen Receptor Type	Key Signaling Pathways	Therapeutic Impact	Clinical Application Prospects	References
Prostate Cancer	ERα、ERβ	1. ERα activates the MAPK/ERK and PI3K/Akt pathways, promoting tumor progression.2. ERβ inhibits cell proliferation, regulates PTEN nuclear translocation, and promotes HIF1α degradation to exert anti-tumor effects.	ERβ agonists (e.g., 8β-VE2) can inhibit cell survival and enhance apoptosis.ERβ can increase chemotherapy sensitivity (e.g., under sunitinib resistance).	Development of selective ERβ agonists for treating highly invasive and castration-resistant prostate cancer.	2,17,32
	GPER	3. GPER activation blocks proliferation and migration, inducing cell cycle arrest.	GPER agonist G-1 can inhibit the growth of castration-resistant prostate cancer cells.	Exploring the potential of GPER agonists in combination therapies for advanced prostate cancer.	
Bladder Cancer	ERα、ERβ	1. ERα induces INPP4B expression to inhibit the AKT pathway, suppressing tumor growth.2. High ERβ expression promotes cell proliferation and invasion by regulating the NF-κB and FOXO1 pathways.	SERMs (e.g., tamoxifen) inhibit ERα and ERβ activity, reducing cell proliferation and invasion. Combining ER modulators with BCG therapy enhances immune efficacy.	Development of personalized therapeutic strategies using SERMs and ERβ inhibitors.	62,137
	GPER	3. GPER activation weakens estrogen-induced tumor proliferation.	GPER agonist G-1 can inhibit cell growth and migration.	Exploring the combination of GPER agonists with immune checkpoint inhibitors to improve therapeutic outcomes.	
Kidney Cancer	ERα、ERβ	1. ERα suppresses DNA repair processes and promotes genomic stability.2. ERβ regulates HIF2α and angiogenesis-related pathways (e.g., ANGPT-2/Tie-2), promoting tumor invasion and angiogenesis.	Selective ERβ inhibitors (e.g., PHTPP) significantly reduce tumor cell proliferation.Combining anti-ERβ agents with targeted TKI drugs improves efficacy.	ERβ modulators combined with anti-angiogenesis therapy may significantly reduce the metastatic potential of kidney cancer.	87,88,97
	GPER	3. GPER activates the PI3K/Akt pathway, promoting migration and invasion.	GPER agonist G-1 improves sunitinib resistance.	Development of combined therapies targeting ERβ and GPER.	
